# Extensive variation between tissues in allele specific expression in an outbred mammal

**DOI:** 10.1186/s12864-015-2174-0

**Published:** 2015-11-23

**Authors:** Amanda J. Chamberlain, Christy J. Vander Jagt, Benjamin J. Hayes, Majid Khansefid, Leah C. Marett, Catriona A. Millen, Thuy T. T. Nguyen, Michael E. Goddard

**Affiliations:** Department of Economic Development, Jobs, Transport and Resources, Agribiosciences Building, 5 Ring Rd, Bundoora, Australia; Dairy Futures Cooperative Research Centre, Agribiosciences Building, 5 Ring Rd, Bundoora, Australia; La Trobe University, Agribiosciences Building, 5 Ring Rd, Bundoora, Australia; Department of Economic Development, Jobs, Transport and Resources, 1301 Hazeldean Rd, Ellinbank, Australia; Institute of Land and Food, University of Melbourne, Royal Parade, Parkville, Australia

**Keywords:** Gene expression, Differential expression, Tissue specific expression, Allele specific expression, Bovine, Cattle, Transcriptomics, RNA sequencing, Regulation

## Abstract

**Background:**

Allele specific gene expression (ASE), with the paternal allele more expressed than the maternal allele or vice versa, appears to be a common phenomenon in humans and mice. In other species the extent of ASE is unknown, and even in humans and mice there are several outstanding questions. These include; to what extent is ASE tissue specific? how often does the direction of allele expression imbalance reverse between tissues? how often is only one of the two alleles expressed? is there a genome wide bias towards expression of the paternal or maternal allele; and finally do genes that are nearby on a chromosome share the same direction of ASE? Here we use gene expression data (RNASeq) from 18 tissues from a single cow to investigate each of these questions in turn, and then validate some of these findings in two tissues from 20 cows.

**Results:**

Between 40 and 100 million sequence reads were generated per tissue across three replicate samples for each of the eighteen tissues from the single cow (the discovery dataset). A bovine gene expression atlas was created (the first from RNASeq data), and differentially expressed genes in each tissue were identified. To analyse ASE, we had access to unambiguously phased genotypes for all heterozygous variants in the cow’s whole genome sequence, where these variants were homozygous in the whole genome sequence of her sire, and as a result we were able to map reads to parental genomes, to determine SNP and genes showing ASE in each tissue. In total 25,251 heterozygous SNP within 7985 genes were tested for ASE in at least one tissue. ASE was pervasive, 89 % of genes tested had significant ASE in at least one tissue. This large proportion of genes displaying ASE was confirmed in the two tissues in a validation dataset.

For individual tissues the proportion of genes showing significant ASE varied from as low as 8–16 % of those tested in thymus to as high as 71–82 % of those tested in lung. There were a number of cases where the direction of allele expression imbalance reversed between tissues. For example the gene *SPTY2D1* showed almost complete paternal allele expression in kidney and thymus, and almost complete maternal allele expression in the brain caudal lobe and brain cerebellum. Mono allelic expression (MAE) was common, with 1349 of 4856 genes (28 %) tested with more than one heterozygous SNP showing MAE. Across all tissues, 54.17 % of all genes with ASE favoured the paternal allele. Genes that are closely linked on the chromosome were more likely to show higher expression of the same allele (paternal or maternal) than expected by chance. We identified several long runs of neighbouring genes that showed either paternal or maternal ASE, one example was five adjacent genes (*GIMAP8*, *GIMAP7* copy1, *GIMAP4*, *GIMAP7* copy 2 and GIMAP5) that showed almost exclusive paternal expression in brain caudal lobe.

**Conclusions:**

Investigating the extent of ASE across 18 bovine tissues in one cow and two tissues in 20 cows demonstrated 1) ASE is pervasive in cattle, 2) the ASE is often MAE but ranges from MAE to slight overexpression of the major allele, 3) the ASE is most often tissue specific and that more than half the time displays divergent allele specific expression patterns across tissues, 4) across all genes there is a slight bias towards expression of the paternal allele and 5) genes expressing the same parental allele are clustered together more than expected by chance, and there are several runs of large numbers of genes expressing the same parental allele.

**Electronic supplementary material:**

The online version of this article (doi:10.1186/s12864-015-2174-0) contains supplementary material, which is available to authorized users.

## Background

There is increasing evidence that many, if not the majority, of mutations that give rise to variation in complex traits reside in regulatory elements that alter gene expression (reviewed by [1]). Mutations in putative regulatory regions have been associated with > 100 phenotypes in human and other species [2, 3] including the classic blond hair phenotype found in northern Europeans ([4], as a result of a variant in the regulatory enhancer of KIT ligand) and stature in cattle ([5], a result of variants in the promoter of PLAG1).

Mutations that affect the expression of an allele on the same chromosome are known as *cis* expression quantitative trait loci (*cis* eQTL). If an individual is heterozygous for such a mutation it is expected that the two alleles of the gene will be expressed unequally causing allelic imbalance or allele specific expression (ASE). This ASE can be detected using RNA sequencing (RNAseq) provided there is a heterozygous site in the coding sequence of the gene. ASE can also be caused by imprinting where an epigenetic mark distinguishes the paternal and maternal chromosomes and causes them to be expressed unequally. Evidence is accumulating from studies in mice and humans that regulatory variation that affects the level of expression, observed as allele specific expression or allelic imbalance, is extremely common. For example Crowley et al. [6] reported that greater than 80 % of mouse genes have *cis* regulatory variation, and estimates from humans and mice range from 4 to 89 % for the proportion of genes showing ASE in at least one tissue (Additional file 1: Table S1).

ASE has been evaluated in a number of studies in humans and mouse, the most extensive being that most recently published by the GTEx consortium [7] in humans. The GTEx consortium used RNAseq data from 29 solid organ tissues, 11 brain sub-regions, whole blood, lymphoblastoid cell lines (LCL) and skin fibroblast cells to study gene expression, including ASE within tissue. They tested for ASE in SNP that were both heterozygous and had greater than 30 RNAseq reads, however they only did this within sample, and found that 1.5–3.7 % of SNP tested showed ASE. Investigating the extent and patterns of ASE in species other than humans and mice, would give insights into the evolution of regulatory variation. Here we report on the extent and pattern of ASE across 18 tissues in a single cow.

Even within humans and mice, let alone other species, there are a number of questions concerning ASE that have only partial answers to date. For instance, to what extent is ASE tissue specific; how often does the direction of imbalance reverse between tissues; how often is only one of the two alleles expressed (mono-allelic expression or MAE); is there a bias towards expression of the paternal or maternal allele; and do genes that are nearby on a chromosome share the same direction of ASE? As we have profiled ASE across so many tissues, our results provide new insights into these questions. As observed for human and mouse, we found pervasive ASE. The majority of ASE was tissue specific. And we also found that more than half of all genes show divergent allele specific patterns across tissues, expressing the paternal allele more highly in one tissue and the maternal allele more highly in another tissue. Further, we found many cases of runs of consecutive genes on a chromosome that were expressed from the same chromosome (paternal or maternal) in a tissue specific manner. Our design does not allow us to distinguish ASE due to *cis* eQTL from that due to imprinting but we report ASE results separately for a group of genes known to be imprinted in cattle, mouse and human and find a complex pattern showing a degree of ASE that varies between tissues.

In order to assess the extent of ASE within and across tissues in a novel outbred species, namely cattle, we collected 18 tissues at a single point in time from one lactating cow. This paper presents results of differences in gene expression between these tissues – the first bovine gene expression atlas using RNASeq data. We then present the discovery of genes showing ASE and present the extent and relationship of this ASE across tissues. ASE was assessed by mapping to parental genomes of the cow sampled, allowing us to minimise any reference bias [8]. We then validate some of these findings in a dataset consisting of two tissues sampled from 20 lactating cows.

## Results and discussion

Between six and 100 million paired sequence reads were generated per library, with three replicate samples for each of the eighteen tissues investigated (Additional file 1: Table S2), resulting in between 47 and 176 million paired sequence reads per tissue. On average 92 % of reads aligned to the reference genome for each library, with greater than 70 % mapping uniquely (Additional file 1: Table S2). Reads mapping to ribosomal RNA (rRNA) genes accounted for less than 0.0001 % of total reads for each sample indicating minimal rRNA contamination during library preparation. Mean coverage of expressed transcripts (5’ to 3’), showed coverage biased toward the 3’ end of transcripts. This is illustrated in Additional file 1: Figure S1 and formally confirmed by a significant regression showing that more reads came from the later exons within a gene (as will be discussed later). This bias is likely a result of some degraded mRNA being sequenced but is small and unlikely to affect subsequent results.

### Tissue specific expression (TSE)

RNAseq reads from each library were aligned to the Ensemble annotation of UMD3.1 bovine reference genome. The total number of expressed genes in each tissue were similar (average 16,935), with mammary gland having the lowest (15,437) and thymus the highest (18,795) number of expressed genes. Within a tissue, the number of reads per gene followed a log-normal distribution (Additional file 1: Figure S2), with many genes contributing close to the average number of reads (10^−5^ of all reads) but a few genes contributing 10 times more or 10 times fewer than the average number of reads. As expected transcripts from mammary gland were dominated by the major milk proteins, with 62 % of transcripts from alpha S1 (*CSN1S1*), alpha S2 (*CSN1S2*), beta (*CSN2*) or kappa (*CSN3*) casein genes, reducing the number of genes observed with low expression. On the other hand thymus was a tissue with high coverage (Additional file 1: Table S2), and this could mean that it had more low abundant transcripts sequenced accounting for the large number of genes expressed.

**Fig. 1:**
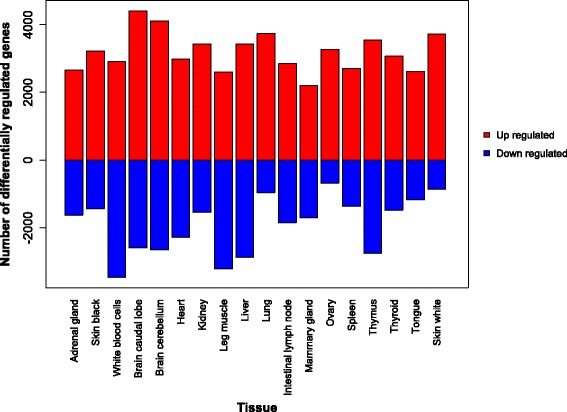
The number of genes up or down regulated with significant (*p* < 0.01) tissue specific expression (TSE) and a greater than two-fold difference in expression, when compared to the average expression of that gene across all other tissues

**Fig. 2:**
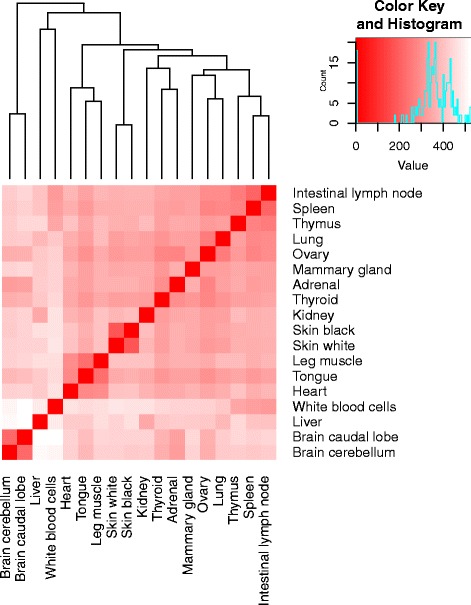
Tissue x Tissue heat map and hierarchical clustering of gene expression data. Colour key indicates the level of expression “relatedness” between tissue types, the darker the colour, the more similar the pattern of gene expression. The histogram in the colour key represents a density plot of the frequency of distance values. The variability in gene expression between tissues is represented by the height of the dendrogram branches

A tissue by gene counts matrix was produced and used to perform differential expression (DE) analysis in which genes that were more expressed in a tissue than in all other tissues (over-expressed), or less often expressed than in other tissues (under-expressed) were identified. With the exception of blood and leg muscle, all other tissues had a greater number of up-regulated genes than down-regulated (Fig. 1). A full list of the genes DE per tissue is contained in Additional file 2: Table S3. When tissues were clustered on their gene expression patterns, using the full normalised count matrix for all genes, tissues were largely grouped together into clusters reflecting their biological relationship, for example muscle tissues clustered together, brain tissues clustered together and skin tissues clustered together (Fig. 2). Liver and blood were both different from the rest of the tissues and each other, as indicated by the height of the dendrogram branches.

Functional annotation of the DE genes in each tissue (Additional file 2: Table S4 and S5) identified many of the biological processes and pathways already reported as having an established role in those particular tissues. For example, in black skin many of the genes such as Keratins, Keratin associated proteins and Dermal allergen genes have known roles in the skin and this was reflected in the enrichment of Gene Ontology (GO) terms for the biological processes ‘epidermis development’, ‘ectoderm development’, ‘hair follicle development’ and ‘keratinization’. The Kyoto Encyclopaedia of Genes and Genomes (KEGG) pathway ‘melanogenesis’, responsible for the deposition of melanin in the skin, is also enriched in the DE genes from black skin. For mammary gland the casein genes, alpha lactalbumin (*LALBA*) and lactoperoxidase (*LPO*) all have well defined roles in the mammary gland. The significantly enriched GO terms ‘epithelium development’, ‘epithelial cell differentiation’, ‘tissue development’ and the KEGG pathway ‘tight junction’, all reflect the mammary gland epithelial cell and tight junction formation, all vital for milk secretion. The GO term ‘defence response to bacterium’ is a term that reflects the need for mammary tissue to constantly fight bacterial infections which can occur through the teat canal.

Counts were also made on an exon basis and this enabled us to ask whether some exons within a gene were more highly expressed than others, and did this vary between tissues? To summarise the sources of variation in transcription the model *y* = *μ* + *tissue* + *replicate* + *tissue. replicate* + *exon*_*number*_. *tissue* + *gene* + *gene. exon* + *gene. tissue* + *exon number* + *error* (see Methods) was fitted, where y = log(read count of each exon within a tissue and replicate), and the variance accounted for by each random effect (*gene, gene.tissue, gene.exon* and *error*) in the model was estimated (Additional file 1: Table S6, and solutions for exon_number_ presented in Additional file 1: Table S7). All fixed effects (*tissue, replicate, tissue.replicate, exon*_*number*_*.tissue*) had highly significant F values. The gene.exon effect accounted for the largest amount of variance (40 %), followed by the gene effect (34 %). The large variance in expression between genes reflects the fact that some genes are highly expressed in all tissues, and others genes are expressed at low levels. The smaller gene.tissue effect (12 % of total variance) reflects differences between tissues in the genes that are highly expressed. The large gene.exon effect is due to the fact that some exons within a gene had much higher counts than other exons within the same gene regardless of tissue, even after accounting for any 3’ bias in coverage of transcripts (Additional file 1: Figure S1) by fitting exon number (order) in the model. This large gene.exon effect implies there could have been a lot of alternative splicing, or alternatively that the annotation of the bovine genome was poor, so that some exons weren’t real or were only occasionally being transcribed. The effect of exon number is due to a bias towards sequencing more exons toward the 3’ end of the gene. This bias varies between tissues (Additional file 1: Table S7), where adrenal gland was the most affected (the most biased) and therefore had the largest effect and mammary and blood were the least affected (least biased) and had the smallest effect of this bias.

### Extent of allele specific expression across tissues

**Fig. 3:**
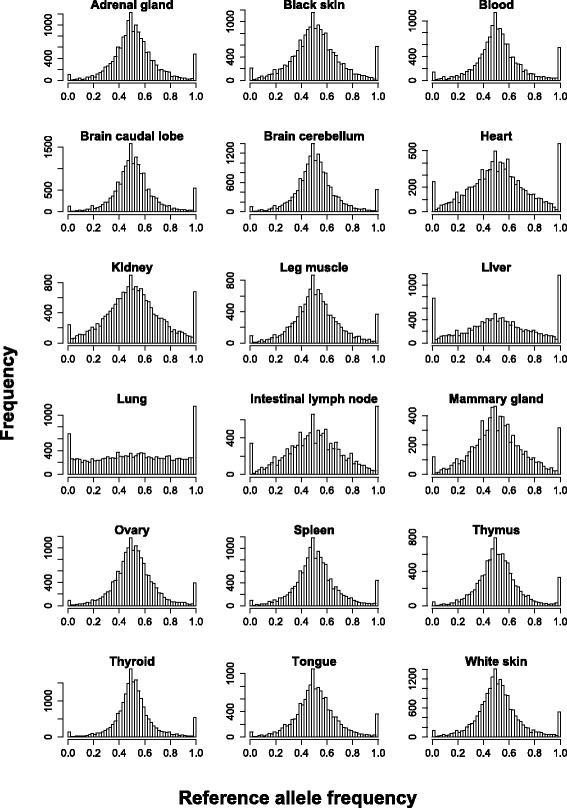
Distribution of reference allele frequency after removing SNP that were likely to be a sequencing error, within tissue type, for all SNP tested for ASE. If there was significant mapping bias still present after mapping to the two parental genomes the distribution of allele frequencies would be skewed toward the reference allele. Instead it followed a normal distribution with a peak at reference allele frequency of 0.5, except for those SNP with reference allele frequency of 0 or 1. As discussed in the text these represent SNP that display monoallelic expression and even though attempts were made to remove SNP that were due to sequencing errors, higher peaks at reference allele frequency of 1, as compared with 0, indicate some still remain

We had access to unambiguously phased genotypes for all heterozygous variants in the cow’s whole genome sequence, where these variants were homozygous in the whole genome sequence of her sire. As a result we were able to map reads to parental genomes [8], to determine SNP and genes showing ASE in the 18 bovine tissues. RNAseq reads from the three replicate libraries were merged and aligned to two parental genomes (see Methods). For all known heterozygous sites that occurred within an exon, allele counts for each alignment were extracted and ASE tested using a Chi-squared test (see Methods) that accounts for any remaining mapping bias. There was very little bias toward the reference allele for all 25,251 SNP tested (Fig. 3). The exception to this was for SNPs that showed mono-allelic expression, it was more often the reference allele than the alternative allele that was expressed (we define MAE as ASE where the frequency of the major allele exceeds 0.9). This could be due to errors in the genome sequence of this cow, with SNP called heterozygous when in fact the cow is homozygous at that position. Such artefacts were also observed by Baran et al. [9] in the 1000 human genome whole genome sequence data and Genotype-Tissue Expression (GTEx) RNAseq data. So the following efforts were made to identify and remove sequencing errors, 1) “private” variant detection was undertaken for this cow, and any SNP that were not discovered in that private detection removed, 2) variants only detected in this cow’s genome and not in any other animal in the 1000 bull genomes project (Daetwyler et al. [10]) were removed, 3) putative runs of homozygosity (RoH) were identified in whole genome sequence data and heterozygous variants falling within those regions were removed using an approach similar to Macleod et al. [11]. After these quality control steps, there was only a slight bias towards expression of the reference allele (mean frequency 0.5014) and this was eliminated when the MAE classes were removed from the data (Fig. 3, Additional file 1: Table S8).

In total 25,251 heterozygous SNP within 7985 genes were tested for ASE in at least one tissue. 89 % of these 7985 genes tested had at least one SNP with significant ASE, in at least one tissue (Table 1). Wang et al. [12] state that genes with multiple SNP supporting ASE have a higher rate of successful verification. Although, a large proportion (49 %) of the genes had only 1 SNP within the gene able to be tested, we found ASE for 3570 of 4856 genes with greater than one SNP tested (i.e. 74 % of genes with more than one heterozygous SNP tested). Therefore we estimate that between 74 and 89 % of genes show ASE in at least one tissue and we conclude that ASE is pervasive in cattle. This estimate is higher than the majority of published literature of 4 to 53 % (Additional file 1: Table S1) from whole genome assessment of ASE. The notable exception is the recent publication from Crowley et al. [6] which reported 89 % of all genes tested in mouse brain showed ASE.

**Table 1:** Allele specific expression analysis results, the number of SNP tested and the number and proportion that showed significant ASE (ASE SNP) in each tissue and in total. Also the number of genes containing SNP tested for ASE (Genes tested) and genes containing greater than one SNP tested for ASE (Genes w/ >1 SNP tested) and then the number and proportion that contained SNP significant for ASE (Genes w/ ASE SNP) and the number and proportion that contained greater than one SNP significant for ASE (Genes w/ >1 ASE SNP) in each tissue and in total. Then finally the number and proportion of genes tested that showed significant ASE in at least one but not all tissues tested (Genes w/ TS ASE SNP) in each tissue and in total

Tissue	SNP tested	ASE SNP (% tested)	Genes tested	Genes w/ >1 SNP tested	Genes w/ ASE SNP (% tested)	Genes w/ >1 ASE SNP (% tested)	Genes w/ TS ASE SNP (% tested)
Adrenal gland	14,698	2636 (18 %)	5462	3134	1635 (30 %)	536 (17 %)	1502 (27 %)
Brain caudal lobe	16,594	2419 (15 %)	5946	3483	1478 (25 %)	494 (14 %)	1318 (22 %)
Brain cerebellum	15,460	2324 (15 %)	5650	3269	1470 (26 %)	466 (14 %)	1337 (24 %)
Heart	9545	2919 (31 %)	3999	2118	1869 (47 %)	618 (29 %)	1768 (44 %)
Intestinal lymph	11,719	3554 (30 %)	4684	2542	2391 (51 %)	782 (31 %)	2284 (49 %)
Kidney	16,616	7442 (45 %)	5925	3457	3958 (67 %)	1751 (51 %)	3750 (63 %)
Leg muscle	11,401	2006 (18 %)	4455	2467	1394 (31 %)	402 (16 %)	1286 (29 %)
Liver	12,507	6773 (54 %)	4887	2715	3574 (73 %)	1612 (59 %)	3381 (69 %)
Lung	14,238	9216 (65 %)	5419	3032	4448 (82 %)	2157 (71 %)	4291 (79 %)
Mammary gland	8161	1543 (19 %)	3566	1838	1100 (31 %)	302 (16 %)	1002 (28 %)
Ovary	15,108	2043 (14 %)	5588	3229	1407 (25 %)	399 (12 %)	1264 (23 %)
Skin black	16,255	4507 (28 %)	5870	3386	2776 (47 %)	999 (30 %)	2608 (44 %)
Skin white	17,087	3533 (21 %)	6004	3531	2156 (36 %)	766 (22 %)	1997 (33 %)
Spleen	14,495	2066 (14 %)	5317	3071	1448 (27 %)	382 (12 %)	1320 (25 %)
Thymus	9781	986 (10 %)	3981	2159	634 (16 %)	182 (8 %)	501 (13 %)
Thyroid	18,181	3279 (18 %)	6196	3703	2013 (32 %)	688 (19 %)	1842 (30 %)
Tongue	12,744	1671 (13 %)	4850	2718	1177 (24 %)	327 (12 %)	1057 (22 %)
White blood cells	12,768	2662 (21 %)	4680	2690	1543 (33 %)	552 (21 %)	1418 (30 %)
Total	25,251	19,082 (76 %)	7985	4856	7067 (89 %)	3570 (74 %)	6631 (83 %)

**Fig. 4:**
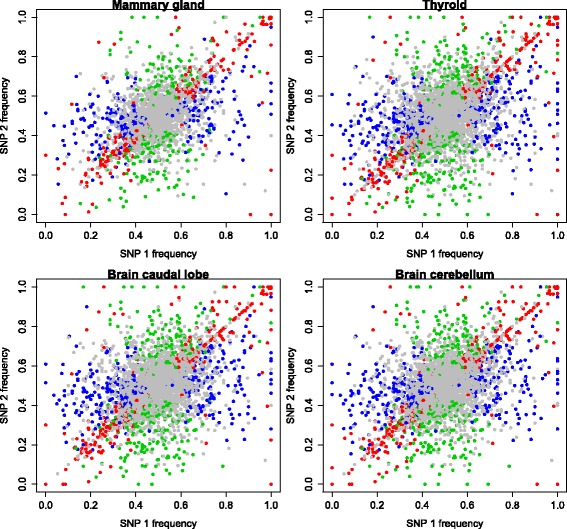
Using only SNP that could be definitively phased and that occurred in genes with 2 or more SNP, these plots for mammary gland, thyroid, brain caudal lobe and brain cerebellum show the paternal allele frequency for neighbouring SNP within a gene. Red points are those where both SNP were significantly ASE, blue points are those where only SNP1 was significantly ASE, green points are those where only SNP2 was significantly ASE, grey points are where neither SNP was significantly ASE

Using only those SNP where parental origin could be definitively determined, and where more than 1 SNP was tested within a gene, we found 72 % overall agreement, up to 82 % in thymus, regarding which allele (paternal or maternal) was expressed between SNP in the same gene (Table 2, Fig. 4), and even better agreement where SNP were in the same exon (Table 2). It is worth noting that lung and liver had the lowest concordance between SNP within the same genes and these are the two tissues with the highest proportion of genes showing ASE (Table 1). It is evident from Fig. 4 that where neighbouring SNP within a gene are both significantly ASE both express the same parental allele, and at very similar frequencies. However it also shows that in a few cases ASE switched between maternal and paternal expression within the gene. This is clearly demonstrated for individual genes in detail in Additional file 1: Figure S3A. Clearly here we can see that some genes showed consistent parental allelic expression across the gene, whereas in other cases the expression changes from one parental allele to another within the gene (Additional file 1: Figure S3A). Such patterns of expression have been previously reported by Wood et al. [13] in human brain and liver and are indicative of allele specific isoform expression. As will be discussed later even the same gene showed different patterns of expression across all 18 tissues types (Additional file 1: Figure S3B, C, D and E).

**Table 2:** Using definitively phased SNP this table shows the number of genes and exons with greater than one SNP in them and the number and proportion of those tested where the same parental allele was expressed, i.e. the SNP were concordant

Tissue	Genes with >1 ASE SNP	Genes with concordant SNP (% tested)	Exons with >1 ASE SNP	Exons with concordant SNP (% tested)
Adrenal gland	347	278 (80.1 %)	224	194 (86.6 %)
Brain caudal lobe	314	358 (66.7 %)	196	230 (74.9 %)
Brain cerebellum	334	249 (76.6 %)	185	173 (86.0 %)
Heart	340	254 (80.8 %)	196	175 (89.2 %)
Intestinal lymph	433	256 (76.6 %)	220	160 (86.4 %)
Kidney	943	241 (70.8 %)	551	156 (79.5 %)
Leg muscle	252	550 (58.3 %)	129	386 (70.0 %)
Liver	872	186 (73.8 %)	524	97 (75.1 %)
Lung	1124	506 (58.0 %)	711	363 (69.2 %)
Mammary gland	187	588 (52.3 %)	100	443 (62.3 %)
Ovary	254	293 (67.6 %)	131	162 (73.6 %)
Skin black	536	147 (78.6 %)	307	82 (82 %)
Skin white	440	199 (78.3 %)	268	108 (82.4 %)
Spleen	260	196 (75.3 %)	126	102 (80.9 %)
Thymus	132	108 (81.8 %)	79	71 (89.8 %)
Thyroid	470	383 (81.4 %)	264	227 (85.9 %)
Tongue	216	174 (80.5 %)	111	96 (86.4 %)
White blood cells	325	327 (74.3 %)	201	215 (91.4 %)

For individual tissues the proportion of genes showing significant ASE varied from as low as 8–16 % of those tested in thymus to as high as 71–82 % of those tested in lung (Table 1). Pant et al. [14] reported 53 % of genes tested showed significant ASE when testing 1389 genes in human white blood cells. Our estimate of 21–33 % of genes tested in white blood cells was lower but we tested more genes (4680, Table 1). Gao et al. [15] reported 30 % of the 8779 genes tested in human mammary epithelial cell lines showed significant ASE, which agrees well with 16–31 % of the 3566 genes tested in mammary gland in this study. Our results in lung indicate that 71–82 % of 5419 genes tested showed significant ASE in this tissue, the highest of any tissue tested. This is much higher than the 2 % of SNP showing ASE estimated by the GTEx Consortium [7], however we tested many more SNP and the GTEx Consortium tested only SNP that were in common across samples and reported only the within sample estimate of ASE. Our estimate of 14–25 and 14–26 % of the 5462 and 5946 genes tested in brain caudal lobe and brain cerebellum respectively are much lower than the estimate of 89 % by Crowley et al. [6] in whole mouse brain, however they had an extremely powerful design testing ASE in greater than 12,000 genes in 96 individuals from all possible pairwise crosses between the three divergent inbred lines, as opposed to the single outbred individual tested here. The power of our study comes from testing many tissues, as did Gregg et al. [16] who tested 52 tissue samples in mouse, all from the mouse brain, and the GTEx Consortium [7] which tested 43 different tissue samples from 175 humans. The GTEx Consortium estimated 1.5 – 3.7 % of SNP tested showed ASE, however they only tested an average of 6385 SNP, a lot less than that tested here, and a lot less than Crowley et al. [6], and their estimates are within sample within tissue and not across all sample and tissues as is reported by Crowley et al. and this study.

**Fig. 5:**
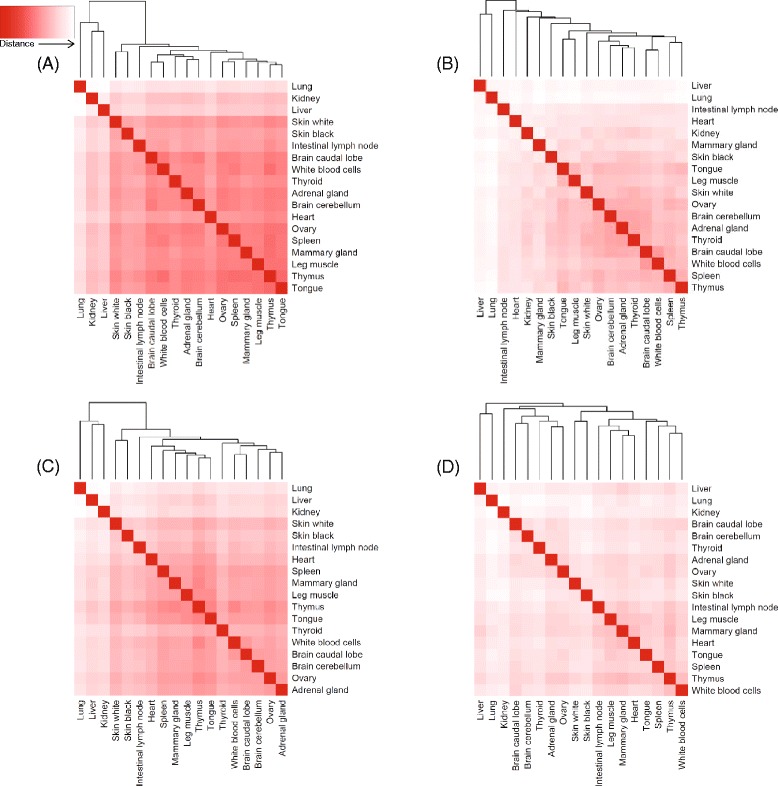
Heat maps of tissue-to-tissue distances generated by applying the ‘dist’ function in R to the transpose of a matrix, as defined for each heat map. For each heat map, the variability between tissues is measured by the height of the dendrogram branches. The colour key indicates the distance between tissues with red being the least distant (or most correlated) and white being the most distant (or least correlated). **a** Uses a SNP by tissue matrix containing the paternal allele frequency of all ASE SNP, therefore displaying the correlation of ASE between tissues. **b** Uses a SNP by tissue matrix in which SNP were assigned “1” if mono-allelic and “0” if not, therefore displaying the correlation of MAE between tissues. **c** Uses a gene by tissue matrix in which genes classified as maternal were assigned “-0.5”, genes classified as paternal were assigned a value of “0.5” and genes that could not be classified or were not expressed were assigned “0”, therefore displaying the correlation of paternal ASE between tissues. **d** Uses a gene by tissue matrix in which all genes in “runs” of 5 or more (as defined in methods) were designated a value of “1” and genes not in a run were designated “0”, therefore displaying the between tissue correlation of genes falling into runs of greater than 5 or more expressing the same allele

**Fig. 6:**
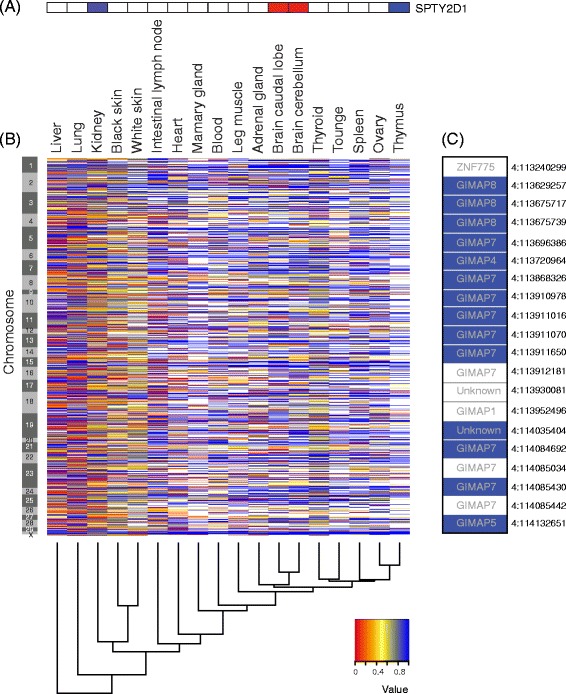
Using only SNP that could be definitively phased, (**a**) is a heat map of the paternal allele frequency of the same SNP on chromosome 29 (in *SPTY2D1*) across all 18 tissues. The frequency of paternal expression is scaled from 0 (100 % maternal, red) to 1 (100 % paternal, blue). **b** A heat map of the frequency of the paternal allele for all phased SNP, for each tissue. Tissues are clustered on the overall paternal expression as indicated by the dendrogram. SNP are ordered by position and the left hand scales indicate the different (ordered) chromosomes. **c** Highlights 14 SNP within 5 known genes (*GIMAP8*, *GIMAP7* copy 1, *GIMAP4, GIMAP7* copy 2 and *GIMAP5,* listed within each cell) on chromosome four in the brain caudal lobe, chromosome and position are listed on the right hand side of the expression pattern

Of the genes showing significant ASE, 94 % displayed ASE in only a subset of the tissues they were expressed in (“Genes w/ TS ASE SNP” in Table 1). Figure 5a demonstrates that ASE was correlated between tissues, where red indicates ASE was correlated, and white indicates it was not. The tissues are clustered based on their ASE and the variability in ASE between tissues is represented by the height of the dendrogram branches. Tissues with similar function, such as black and white skin, cluster together as they did with gene expression (Fig. 2). However some ASE relationships are different to what they were for gene expression, i.e., heart does not cluster with the other muscle samples for ASE, while it did for gene expression, indicating tissue specific differences in ASE. Interestingly lung, liver and kidney show very little correlation of ASE with any other tissues or with each other, these tissues showed the highest levels of ASE. Of the 4033 genes expressed in more than one tissue showing ASE, and where the major allele’s parental origin could be determined (see Methods), 2324 (58 %) genes showed divergent allele specific patterns, that is the major allele expressed was not the same across all tissues. This was previously demonstrated in Additional file 1: Figure S3A. An example of a single SNP is given in Fig. 6a for a SNP in gene *SPTY2D1*, where the paternal allele is expressed in kidney and thymus (0.82 and 0.92 paternal allele frequency respectively) and maternal allele in the brain caudal lobe and brain cerebellum (0.12 and 0.08 paternal allele frequency respectively). Mutations in *SPTY2D1* (Suppressor of Ty, domain containing 1) have been associated with lipid levels in humans in multiple studies [17, 18], and interestingly Guo et al. [18] suggested this association might be sex specific. Additional file 1: Figure S3B, C, D and E shows examples of ASE patterns across tissues for multiple SNP within the genes *GBP5*, *PRUNE2* , *SGOL2* and *SAMD9.* They demonstrate that allele specific expression patterns within genes and across tissues can be extremely divergent and complex, when looking at multiple SNP in many tissues.

Tissue specific ASE (TS ASE) has been reported previously. One of the most extensive tests, by Keane et al. [19], performed RNAseq in a single F1 mouse and tested 6975 genes across 6 tissues and found that 95 % of genes showing ASE did so in a tissue specific manner. Another by Pinter et al. [20] performed RNAseq in a reciprocal cross between two mouse strains, testing 7465 genes across two tissues and found 82 % of ASE genes showed TS ASE. Keane et al. [19] and Pinter et al. [20] also found that 12 and 21 % of ASE genes showed divergent allele specific patterns.

Interestingly we found that there are ten genes that show ASE exclusively in mammary gland, ZMYND11, CLSPN*, SLC30A2*, TECTB*, SERTAD2, ZNF638, ALDH3B2*, CIDEA*, TFF1* and one uncharacterized protein*. Seven of these genes are differentially expressed when compared with all other tissues (denoted with * above) and all seven are up regulated. These genes have functions associated with mammary development, involution, or milk nutrition; *ZMYND11*, *CLSPN*, *TFF1*, affect proliferation or apoptosis of mammary cells [21–23]; *SLC30A2* transports Zinc from the mammary gland into milk [24]; *CIDEA* is a transcriptional coactivator regulating mammary gland secretion of milk lipids [25]; and *TFF1* is a gastrointestinal protective peptide secreted into milk [26]. Genes that show ASE exclusively in one tissue are often the genes that are differentially expressed, when comparing that tissue with all other tissues (Additional file 1: Table S9). This is a theme which is consistent across all tissues (Additional file 1: Table S9). This could simply be because we have greater power to detect ASE in highly expressed genes. However, it may also be that genes that are highly expressed in a particular tissue are likely to be regulated by multiple sites in the surrounding DNA and these sites are likely to contain at least one SNP which is heterozygous. All genes that show exclusive TS ASE are listed in Additional file 2: Table S10. There were some highly expressed genes that did not show significant ASE, such as alpha s2 casein (*CSN1S2*) and beta casein (*CSN2*) which are two of the major milk proteins highly expressed in mammary gland (Additional file 1: Table S11) , indicating that our test does not always find ASE in highly expressed genes.

Monoallelic expression (MAE) in at least one tissue was observed in 4298 of 7985 genes tested (54 %) and for 1349 of 4856 genes tested with more than one heterozygous SNP with ASE (28 %, Table 3). We have categorised ASE as MAE where the major allele is at a frequency >90 %. This category of genes is the most affected by errors in the cow’s genome sequence (i.e. Fig. 3) and so we consider the 28 % of genes with MAE for more than one SNP to be most likely to be MAE (as opposed to genes with MAE for just a single SNP), but that the true estimate is somewhere between 28–54 %. Within tissue estimates of genes that are MAE in this study range from 4.1–5.8 % in thymus and thyroid and 24–38 % in lung with an average of 7–12 % across all tissues. The correlation of MAE between tissues (Fig. 5b) shows some correlation between their MAE patterns but largely these expression patterns are more different between tissues than ASE (compare Fig. 5b and a). Our conservative estimate of 28 % of genes showing MAE across tissues is higher than reported by Keane et al. [19] in 6 mouse tissues who found that 12 % of genes showed significant extreme (defined as >75 % major allele) allelic bias and Pinter et al. [20] who found that 5 % of genes tested showed monoallelic expression (defined as 100 % major allele) in 2 mouse tissues. However in this study we have tested many more tissues than either of those studies. Our estimates of 7–12 % within single tissues agree with published reports - Gimelbrant et al. [27] reported that 5–10 % of 4000 tested autosomal genes displayed random monoallelic expression in human clonal cell lines, and Zwemer et al. [28] report that 10 % of 1358 autosomal genes tested showed random monoalleleic expression in mouse cell lines. Our estimates of 7–12 % within single tissues agree with these published reports.

**Table 3:** Monoallelic expression results, the number and proportion of SNP tested that were showing monoallelic expression (MAE SNP), that is the major allele is at a frequency >90 %, in each tissue and in total. Also the number and proportion of genes tested that contained MAE SNP (Genes w/ MAE SNP). Then the number and proportion of genes with greater than one SNP showing MAE (Genes w/ 1 MAE SNP) in each tissue and in total

Tissue	MAE SNP (% tested)	Genes w/ MAE SNP (% tested)	Genes w/ >1 MAE SNP (% tested)
Adrenal gland	690 (5 %)	370 (6.8 %)	144 (4.6 %)
Brain caudal lobe	776 (5 %)	398 (6.7 %)	164 (4.7 %)
Brain cerebellum	665 (4 %)	340 (6.0 %)	139 (4.3 %)
Heart	994 (10 %)	702 (17.6 %)	197 (9.3 %)
Intestinal lymph	1236 (11 %)	881 (18.8 %)	237 (9.3 %)
Kidney	1362 (8 %)	859 (14.5 %)	266 (7.7 %)
Leg muscle	564 (5 %)	361 (8.1 %)	106 (4.3 %)
Liver	2333 (19 %)	1540 (31.5 %)	484 (17.8 %)
Lung	3215 (23 %)	2080 (38.4 %)	726 (23.9 %)
Mammary gland	544 (7 %)	373 (10.5 %)	108 (5.9 %)
Ovary	603 (4 %)	349 (6.2 %)	128 (4.0 %)
Skin black	1032 (6 %)	680 (11.6 %)	186 (5.5 %)
Skin white	870 (5 %)	501 (8.3 %)	173 (4.9 %)
Spleen	627 (4 %)	372 (7.0 %)	126 (4.1 %)
Thymus	429 (4 %)	230 (5.8 %)	89 (4.1 %)
Thyroid	750 (4 %)	361 (5.8 %)	153 (4.1 %)
Tongue	531 (4 %)	321 (6.6 %)	108 (4.0 %)
White blood cells	803 (6 %)	401 (8.6 %)	171 (6.4 %)
Total	8546 (34 %)	4298 (53.8 %)	1349 (27.8 %)

### Validation of allele specific and mono allelic expression

In an attempt to confirm some of the above findings we tested ASE in a validation dataset. This data consisted of RNA sequence reads from 20 first lactation Holstein dairy cows for two tissues, liver and white blood cells (WBC). The data was mapped and tested for ASE in a similar manor to the original dataset above (see Methods for details). However these animals did not have whole genome sequence data and so greater than 28 million SNP were imputed for these animals and phased genotypes used to create parental genomes and then tested for ASE in the RNA sequence reads. On average 19 million reads were generated per library with 82.8 % passing quality control, and 89 % of those mapped uniquely to both the maternal and paternal genomes (Additional file 1: Table S12).

On average, across the 20 samples, 13–29 % of the 3531 genes tested in white blood cells and 12–26 % of the 2939 genes tested in liver showed significant ASE (Additional file 1: Table S13). However across all samples 35–72 % of 6521 genes tested in white blood cells and 31–65 % of 8187 genes tested in liver showed significant ASE (Additional file 1: Table S13). That means that 72 and 65 % of all genes tested across all samples showed significant ASE in white blood cells and liver respectively, in at least one animal. The within sample estimates in white blood cells agreed very well with the 21–33 % of genes that showed ASE in the discovery dataset (Table 1). Within sample estimates in liver were dramatically lower than that of the 59–73 % of genes that showed ASE in the discovery dataset (Table 1). However on average samples in the validation dataset had only 15 million paired reads mapped and tested only 2939 genes, compared with the discovery dataset that had 102 million paired reads mapped and tested 4887 genes for liver, therefore this reduction in the proportion of genes showing ASE is likely a reflection of the reduced sequence coverage and therefore power to detect ASE in individuals within the validation dataset.

On average, across all 20 samples, 0.6–4.3 % of 3531 genes and 0.7–4 % of 2939 genes tested for white blood cells and liver respectively showed significant MAE (Additional file 1: Table S14). However across all samples 2.5–22 % of 8970 genes and 2.6–20 % of 8187 genes tested in white blood cells and liver respectively showed significant MAE respectively (Additional file 1: Table S14). These within sample estimates were much lower than the 6.4–8.6 and 17.8–31.5 % of genes tested in white blood cells and liver respectively of the discovery dataset. However again we attribute this to the sequence coverage and therefore the lower power to detect ASE (including MAE) in the individual validation samples.

Interestingly when we combined samples within the validation dataset the proportion of genes that showed ASE increased to 35–72 and 31–65 % in white blood cells and liver respectively (Additional file 1: Table S13). Also the proportion of genes that show MAE increased to 2.5–22 and 2.6–20 % in white blood cells and liver respectively (Additional file 1: Table S14). This reflects the variation in the genes that show ASE across the 20 animals (Additional file 1: Figure S4) and the moderate correlation between individuals (within tissue) of genes showing ASE (Additional file 1: Figure S5). From Additional file 1: Figure S4 we can see that there are only 1847 of the 6298 genes that showed ASE (Additional file 1: Table S14) in five or more of the 20 animals in WBC, and 1248 of the 5169 genes in liver. These drop to 519 and 370 for WBC and liver respectively in ten or more of the samples. For the validation dataset only, the correlation of genes showing ASE is higher within tissues (Additional file 1: Figure S5), however the correlations are still only moderate, reflecting the variability in genes that display ASE across multiple individuals. Additional file 1: Figure S5 also shows that when the same tissue samples from the discovery dataset are included, the two tissues from the discovery dataset are more correlated with each other than they are with the other samples from the same tissues. We hypothesis that this difference is a reflection of the number of genes able to be used in the correlation calculation, therefore the power of the test is greater for that comparison. This relationship was also reported by the GTEx consortium [7]. They compared between-sample, and between-tissue, sharing of ASE with overall similarity of gene expression. They found that gene expression levels were similar between individuals when comparing within tissue, and different between tissues when comparing within individual gene expression, as you would expect. However allelic ratios had a higher correlation among tissues from the same individuals than among different individuals for the same tissue, suggesting that ASE is primarily determined by the individual’s genome. Therefore by testing only one individual we have likely underestimated the total number of genes displaying ASE in the cattle population, and further testing in more individuals will uncover more genes that show ASE.

### Preferential expression of the paternal allele

Using the SNP with known parental origin, we were able to classify genes as expressing either the paternal or maternal allele. Our results found that on average 54.17 % of all genes within a tissue, with significant ASE SNP and where parental origin could be determined, favoured expression of the paternal allele (Table 4). The proportion of genes expressing the reference allele was on average 50.76 %, a difference of 3.41 %. Using a t-test we found there was significantly (*p* = 0.04) more paternal expression than reference allele expression (with pairs being tissues). The distribution of all genes where parental origin could be determined (not just those containing SNP significantly ASE) is depicted in Additional file 1: Figure S6. It shows that on average across all tissues 13.3 % of all genes tested express both alleles equally, 39.7 % express the maternal allele preferentially and 46.9 % express the paternal allele preferentially. This can also be seen in the heat map of allele expression (Fig. 6b), where the majority of tissues are dominated by paternal allele expression (blue). The tissues are also clustered on the x axis by the alleles that are expressed, so that the tissues on the left have more maternal expression (yellow) but, as we move across the tissues to the right, the amount of maternal expression decreased. However when SNP with paternal allele frequency of zero or one were removed, 50.74 % of all genes favoured expression of the paternal allele. This indicates that it is the genes that show MAE that are favouring the paternal allele. The correlation of this paternal expression between tissues is shown in Fig. 5c, which demonstrated correlation between tissue’s paternal ASE patterns, but largely these expression patterns are more different between tissues than ASE (Fig. 5a). Crowley et al. [6] reported that 54–60 % of all genes, with significant ASE, favoured expression of the paternal allele in mouse brain. This is consistent with our estimates of 53–57 % in brain caudal lobe and in brain cerebellum respectively (Table 4). Because we were only able to determine phase in ~50 % of the SNP tested we have not been able to test as many genes as was tested by Crowley et al. [6]. We have however tested many more tissues and found the same allele expression pattern favouring the paternal allele in many tissues (Table 4).

**Table 4:** The total number of genes showing significant ASE, where phase could be determined, and the number and proportion of those where the major allele is the paternal allele and the reference allele

Tissue	ASE genes classified	Paternal genes (%)	Reference genes (%)	WW runs test (*p*-value)
Adrenal gland	1189	594 (49.95 %)	647 (54.41 %)	0.0006*
Brain caudal lobe	1038	558 (53.75 %)	530 (51.05 %)	0.54
Brain cerebellum	1026	582 (56.72 %)	497 (48.44 %)	0.0063*
Heart	1360	710 (52.20 %)	702 (51.61 %)	0.27
Intestinal lymph	1645	873 (53.06 %)	862 (52.40 %)	0.40
Kidney	3490	1807 (51.77 %)	1718 (49.22 %)	0.001*
Leg muscle	910	473 (51.97 %)	457 (50.21 %)	0.5
Liver	3236	1672 (51.66 %)	1633 (50.46 %)	0.18
Lung	4321	2286 (52.90 %)	2179 (50.42 %)	0.04*
Mammary gland	721	370 (51.31 %)	346 (47.98 %)	0.66
Ovary	854	488 (57.14 %)	422 (49.41 %)	0.09
Skin black	2058	1114 (54.13 %)	1048 (50.92 %)	0.009*
Skin white	1587	889 (56.01 %)	809 (50.97 %)	0.018*
Spleen	897	513 (57.19 %)	436 (48.60 %)	0.27
Thymus	398	240 (60.30 %)	221 (55.52 %)	0.11
Thyroid	1483	808 (54.48 %)	753 (50.77 %)	0.00018*
Tongue	749	437 (58.34 %)	365 (48.73 %)	0.11
White blood cells	1101	575 (52.22 %)	579 (52.58 %)	0.03*
Average		54.17 %	50.76 %	

Note phase could not be determined for all SNP tested. Also *p*-value from the Wald-Wolfowitz (WW) runs test in each tissue, where * denotes those that are significant

There is now accumulating evidence that expression within cells is from one chromosome (paternal or maternal). Borel et al. [29] tested ASE in 203 single cells. They found that for most actively transcribed genes one allele was predominantly expressed in each of the single cells, and that equal numbers of single cells expressed one or the other allele, with very few (less than 5 % of cells) expressing both alleles. Pinter et al. [20] performed RNA-DNA FISH and RNAseq on mouse tail fibroblast cells. RNA-DNA FISH showed that the majority of single cells expressed only a single allele. Also, for genes that showed ASE in the RNAseq data, there was an imbalance in the number of cells expressing the major allele. So if this is the case in all mammals then what we see in this study is that within certain tissues there are more cells within a tissue complex expressing the paternal allele at any one time for genes that show MAE.

### Coordinated expression of neighbouring genes

To investigate whether there was evidence for co-expression of genes in close proximity to each other, we performed a Wald-Wolfowitz runs test on an ordered list of genes classified by paternal or maternal expression in each tissue (Table 4). Our results show that nine of the 18 tissues displayed significant (*p* < 0.05) clustering of expression, greater than expected by chance. This means that neighbouring genes were more likely to express the allele from the same chromosome (maternal or paternal). Figure 5d shows the correlation between tissues of clusters with greater than five genes expressing the same allele and Additional file 1: Figure S7 shows the frequency of runs of 2–40 genes showing the same parental inheritance. For brain caudal lobe, one chromosome region showing expression from the same chromosome included 14 SNP across the five known genes, *GIMAP8*, *GIMAP7* copy1, *GIMAP4*, *GIMAP7* copy 2 and *GIMAP5* that all detect strong ASE of the paternal allele (Fig. 6c). These five known genes are from one gene family known as GIMAP family (GTPase, IMAP Family) and they have no known common control region. The function for these genes in brain is not known but previous studies have found these genes predominantly expressed in mature lymphocytes, and suggest they have a role in lymphocyte survival [30]. Crowley et al. [6] also found that genes with higher expression from one parental allele tended to cluster in mouse. They found that, among the 19 autosomes, 15 had a higher proportion of genes whose neighbour had the same parental skew in expression which was more than expected by chance.

### Allele specific expression in known imprinted genes

One source of ASE is imprinting, so we investigated whether genes previously found to be imprinted in mammals (humans, mice or cattle), extracted from the gene imprint database (http://www.geneimprint.com/site/home), showed extreme ASE in our data set. Thirty one of these genes, four of which had been previously reported to be imprinted in cattle (*NAPIL5, SLC22A3, RTL1, Igf2r*), could be tested for imprinting in our data because there was both expression of the gene (at least 10 reads) and a heterozygous SNP in the transcripts from that gene. Genes were classified as either not imprinted, imprinted, partially imprinted, tissue specifically imprinted or tissue specific partially imprinted (Table 5). There was evidence for ASE in 17 genes out of the 31 tested, including all four previously reported as imprinted in cattle (Table 5). Twelve of the 17 genes showed ASE in specific tissues, varying from one of the tissues showing expression (e.g., *COPG2IT1*), to all but one of the tissues showing expression (e.g., *IGF2*) (Table 6 and Additional file 1: Table S15). Six of the 17 genes had the same allele expressed as was previously reported, including all 4 genes previously reported as imprinted in cattle (Table 6). Seven had the opposite allele expressed but none of these genes had previously been reported as imprinted in cattle (Table 6). For example Adenosine monophosphate deaminase 3 (*AMPD3*) was reported as expressing the maternal allele in mice, however our data suggests tissue dependant ASE. Here we found the paternal allele expressed in thyroid and blood, and the maternal allele expressed in lung. *Bos Taurus* growth factor receptor-bound protein 10 (*GRB10*) imprinting was reported as isoform dependant in human and mouse, here in cattle we found partial imprinting of just the paternal allele in all 11 tissues.

**Table 5:** Summary of classification of genes previously found to be imprinted in human and mice or cattle

Class	ASE	MAF >90 %	All tissues	Human & mouse	Cattle
Not Imprinted	No	No	Yes	14	0
Imprinted	Yes	Yes	Yes	2	2
Partially Imprinted	Yes	No	Yes	1	0
Tissue Specifically Imprinted	Yes	Yes	No	3	2
Tissue Specific Partially Imprinted	Yes	No	No	7	0
Total tested				27	4
Total imprinted				13	4

The number of genes classified as either not imprinted, imprinted, partially imprinted, tissue specifically imprinted or tissue specific partially imprinted. ASE defines whether the class of genes showed significant ASE, MAF >90 % defines if the major allele frequency was greater than 90 % and All tissues defines if the ASE result was observed across all tissue the gene was expressed in

**Table 6:** Genes found to be imprinted in this dataset, along with gene identifiers, which species and which allele was previously reported, the number of SNP used to test for imprinting, the number of tissues the SNP were expressed in, the number of tissues where the SNP showed imprinting or partial imprinting, the imprinting status and expressed allele in this dataset. The expressed allele was determined from the SNP tested or where they were not informative from other SNP within the gene but not within exons or from SNP flanking the gene

Bovine ensembl ID	Gene name	Expressed allele reported	Species reported	#SNP	#Tissues expressed	#Tissues imprinted	#Tissues partially imprinted	Imprinting status	Expressed allele
ENSBTAG00000010128	*NAP1L5*	Paternal	human, mouse, cattle	3	13	13	0	I	
ENSBTAG00000018645	*DLX5*	Maternal	human	1	1	1	0	I	Paternal
ENSBTAG00000039080	*SLC22A3*	Maternal	human, mouse	1	5	5	0	I	Maternal
ENSBTAG00000046585	*RTL1*	Paternal	human, mouse, cattle	3	1	1	0	I	Paternal
ENSBTAG00000047473	*NLRP2*	Maternal	human	8	1	0	1	PI	Paternal
ENSBTAG00000002402	*Igf2r*	Maternal	mouse, cattle	2	14	4	1	TSI	Maternal
ENSBTAG00000008361	*Pon2*	Maternal	mouse	3	14	0	1	TSPI	Paternal^a^
ENSBTAG00000013066	*Igf2*	Paternal	mouse, cattle	1	13	12	0	TSI	Paternal
ENSBTAG00000017245	*COPG2IT1*	Paternal/Maternal	human, mouse	1	14	1	0	TSI	Paternal^a^
ENSBTAG00000024426	*PPP1R9A*	Maternal	human, mouse	2	9	2	0	TSI	Paternal
ENSBTAG00000027081	*ATP10A*	Maternal	human, mouse	13	9	0	4	TSPI	Paternal^a^
ENSBTAG00000034645	*Pon3*	Maternal	mouse	1	13	5	0	TSI	Paternal^b^
ENSBTAG00000002813	*Gab1*	Paternal	mouse	2	18	0	1	TSPI	
ENSBTAG00000003035	*Impact*	Paternal	mouse	18	18	0	17	TSPI	Paternal
ENSBTAG00000006640	*RB1*	Maternal	human	4	18	0	4	TSPI	Paternal^ab^
ENSBTAG00000015821	*Ampd3*	Maternal	mouse	6	12	0	3	TSPI	Tissue Specific^c^
ENSBTAG00000017086	*GRB10*	Isoform dependant	human/mouse	8	16	0	11	TSPI	Paternal

^a^Based on SNP within gene boundaries but not within exon

^b^Based on SNP upstream of gene

^c^Based on SNP up and downstream of gene. Lung maternal, thyroid and lymph node paternal

Imprinting status is defined as: *I* Imprinted, *PI* Partially imprinted, *TSI* Tissue specifically imprinted, *TSPI* Tissue specific partially imprinted

Because we could distinguish maternal and paternal alleles only in one animal, we were unable to distinguish imprinting from *cis* eQTL in this study. However, it seems likely that the ASE we observed in the four genes previously reported to be imprinted in cattle (*NAPIL5, SLC22A3, RTL1, Igf2r*) is due to imprinting. The results for other genes could be due to *cis* eQTL or they may indicate that imprinting is a more variable phenomenon than classically described and varies in extent and tissue specificity.

### Implications for cattle breeding

Our attention must now turn to the identification of these *cis*-regulatory variants, and the international FAANG (Functional Annotation of Animal Genomes) consortium [31] aims to do this for livestock species. The identification of causative regulatory variants could then be used in livestock genomic selection programs leading to more accurate genomic breeding values and increases in the rate of genetic gain for economically important traits in cattle and other livestock species. A promising example where this has been tried has been in chicken’s response to Mareks disease infection, [32, 33]. The authors discovered SNP that showed ASE in response to infection and found they accounted for ~83 % of the genetic variance for Mareks disease resistance [32]. They then used those SNP to genotype a resource population of 1000 chickens to generate SNP effects for Mareks disease resistance, and used those in the genomic prediction of 60 roosters. These roosters were progeny tested for Mareks disease resistance. The genomic predictions based on the ASE SNP had 61 % higher accuracy than traditional breeding values [33]. So although the SNP used were unlikely to be causative they would have been in high LD with the causative regulatory variants, resulting in them being highly predictive of Mareks disease resistance. This approach could be used in cattle for important diseases such as tuberculosis and trypanosomiasis. The identification of causative regulatory variants is expected to further increase the accuracy of genomic predictions for production and health traits.

## Conclusion

Investigating the extent of ASE across 18 bovine tissues in one cow and two tissues in 20 cows demonstrates 1) ASE is pervasive in cattle, 2) the ASE is often MAE but ranges from MAE to slight overexpression of the major allele, 3) the ASE is most often tissue specific and that more than half the time displays divergent allele specific expression patterns across tissues 4) the expression slightly favours the paternal allele and 5) genes expressing the same parental allele are clustered together more than expected by chance, and there are several runs of large numbers of genes expressing the same parental allele.

## Methods

### Tissue sampling

Eighteen tissues: black skin, white skin, adrenal gland, brain caudal lobe, brain cerebellum, heart, kidney, liver, lung, intestinal lymph node, mammary, leg muscle (semimembranosus), ovary, spleen, thymus, thyroid and tongue, from one lactating dairy cow were collected directly after euthanasia, whole blood was collected by venipuncture of the coccygeal vein just prior to euthanasia. The cow was 25 months old and 65 days into her first lactation and was euthanized because she injured her rear leg not for the purposes of this study, for this reason the local Animal Ethics Commitee (DEPI Agricultural Research and Extension Animal Ethics Commitee) advised ethics approval was not required. Tissues were dissected by a veterinarian and then into ~100 mg tissue samples and snap frozen in liquid nitrogen. Tissues were stored at -80oC. Blood was processed according to the blood fractionation and white blood cell stabilisation procedure in the RiboPure™ blood kit (Ambion by Life Technologies) protocol. White blood cells in RNA later were then stored at -20oC.

### RNA extraction, library preparation and sequencing

One hundred mg of each tissue was ground in triplicate using a TissueLyserII (Qiagen) and liquid nitrogen. RNA was extracted from ~30 mg of ground tissue using Trizol (Invitrogen) according to standard protocol. RNA was then passed through an RNeasy column (Qiagen) and eluted in 30ul RNase free water. RNA was extracted from stabilised white blood cells using RiboPure™ blood kit according to protocol. RNA quality was assessed on BioAnalyser 2100 (Agilent Technologies). RNAseq libraries were prepared using the TruSeq RNA sample preparation kit (Illumina) according to the protocol. Three multiplexes of 12 libraries and one multiplex of 6 libraries, each with one of the 12 indexed adaptors, were pooled. Each pool was sequenced on one flowcell lane on the HiSeq2000 sequencer (Illumina) in a 101 cycle paired end run. 100 base paired end reads were called with CASAVA v1.8 and output in fastq format. Sequence quality was assessed using FastQC. In house scripts were used to trim and filter poor quality bases and sequence reads. Bases with quality score less than 20 were trimmed from the 3’ end of reads. Reads with mean quality score less than 20, or greater than 3 N, or final length less than 50 bases were discarded. Only paired reads were retained for alignment.

### Tissue specific expression (TSE) analysis

Paired RNA reads were aligned to the Ensembl annotation release 75 of bovine genome assembly UMD3.1 using TOPHAT2 [34]. Bam files are available from NCBI Sequence Read Archive and can be found using study accession number SRP042639. Custom scripts were used to assess sequencing performance, library quality and alignment quality. RNA-seqQC [35] was used to perform gene body plots: plot of the mean coverage for expressed transcripts from the 5’-3’ end, with the lengths of transcripts normalised to 1–100. The python package HTSeq [36] was used to generate a tissue by gene and a tissue by exon count matrix from all TOPHAT2 alignment files. TSE was analysed using the R software package edgeR [37] defining a design matrix for which the intercept was the overall mean gene expression. A gene was defined as having TSE if it was significantly (*p* < 0.01) differentially expressed and if its expression level in a tissue was greater than two-fold different to the average expression of that gene across all other tissues. Hierarchical clustering of TSE was performed using the R software packages DESeq [38] and gplots (http://cran.r-project.org/web/packages/gplots/).

To determine the proportion of reads from each gene contributing to the transcriptome in each tissue we calculated and plotted the frequency of the proportion of RNA molecules from each gene (*i*) as $RN{A}_i=\frac{Count{s}_i+10}{Lengt{h}_i\times T}$, where $T={\displaystyle \sum_{gene s}}\frac{Count{s}_{gene}+10}{Lengt{h}_{gene}}$ and *Length* was the sum of the length of all exons for gene.

To determine where the variation in transcription was occurring the model *y* = *μ* + *tissue* + *replicate* + *tissue. replicate* + *exon*_*number*_. *tissue* + *gene* + *gene. exon* + *gene. issue* was fitted, where *y* = ln(1 + *count*_*e*_), μ was mean, *tissue* was the tissue effect, *replicate* was the tissue replicate effect, *gene* was gene effect, *exon*_*number*_ was exon effect where exon’s are ordered as a fraction of the total exon number and *count*_*e*_ was the read count per exon. *tissue*, *replicate*, *tissue.replicate* and *exon*_*number*_*.tissue* were fitted as a fixed effect to correct for different average coverage per sample (and three samples per replicate). The variance components were then estimated using R.

Functional annotation of the top 200 most significant differentially expressed genes in each tissue was performed using the Database for Annotation, Visualisation and Integrated Discovery (DAVID) v6.7 [39].

### Allele specific expression (ASE) analysis

As a part of the 1000 bull genomes project [10] this cow’s whole genome was sequenced to an average fold coverage of 44 (NCBI Sequence Read Archive accession number SRX527665) then SNP and their corresponding phased genotypes were called using an in house pipeline (see [10], for methods). The phased SNP genotypes from Run 3.0 of the 1000 bull genomes project were used in this study to create two parental genomes. For each parental genome, at each SNP position in the UMD3.1 reference, the reference allele was replaced with the allele from the corresponding haplotype. Paired RNA reads were then aligned to each of the parental genomes using TOPHAT2 [34] allowing for two mismatches. Each set of parental alignments were treated individually in the following manner: For each tissue, accepted_hits.bam files from the three replicates were merged, sorted and indexed using SAMtools [40]. Using the list of known heterozygous SNP from the 1000 bull genomes project and using only those that fell within the gene exon boundaries as dictated by Ensembl release 75 annotation of UMD3.1 bovine genome assembly, the SAMtools mpileup function (version 0.1.14) was used to extract allele counts for each SNP. The perl vcfutils.pl varFilter tool was then used with “-Q 0 -a 0” options to filter the summary VCF file. The 1000 bull genomes pipeline was then also used to perform “private” SNP detection for this cow, using only her whole genome sequence and not any other animals in the 1000 bull genomes project. SNP were then filtered to only consider those that were detected in the “private” variant detection, had a read depth of at least 10 in both parental haplotype alignments and the most abundant allele in the maternal genome was the most abundant allele in paternal genome. This last rule was used to remove regions with obvious mapping bias to the reference genome. SNP were considered as having significant ASE where they had a Chi-squared (*χ*^2^) value corresponding to *p* < 0.01,

$$ {\chi}^2=\frac{\left(\frac{{\left({r}_m{a}_p-{a}_m{r}_p\right)}^2N}{ramp}\right)}{2} $$

Where *r* was the count of reference alleles aligned to both the maternal and paternal genomes, *a* was the count of alternate alleles aligned to both the maternal and paternal genomes, *m* was the count of reference and alternate alleles aligned to the maternal genome, *p* was the count of reference and alternate alleles aligned to the paternal genome, *r*_*m*_ was the count of reference alleles aligned to the maternal genome, *r*_*p*_ was the count of reference alleles aligned to the paternal genome, *a*_*m*_ was the count of alternate alleles aligned to the maternal genome, *a*_*p*_ was the count of alternate alleles aligned to the paternal genome and *N* was the total number of alleles aligned to the maternal and paternal genomes. The traditional Chi-squared value was divided by two to account for the value of *N* being derived from the counts of both parental haplotypes. ASE SNP with a major allele frequency (averaged from both parental genome alignments) greater than or equal to 0.9 were considered to have monoallelic expression (MAE). Because sequence errors would also result in MAE we conducted further filtering of ASE SNP, removing 1) SNP that were not heterozygous in any other animal in the 1000 bull genomes Run3.0 dataset, 2) SNP that fell within regions of the cows genome sequence deemed to be homozygous. Regions of homozygosity were determined by stepping through the genome 50 kb at a time examining a window of 100 kb (i.e. 100 kb windows offset by 50 kb tiles). The number of heterozygous SNP falling in each window was counted and if less than 6, the region was flagged. If the windows immediately adjacent to flagged regions were also flagged, then the regions were deemed to be homozygous.

Using the sire’s genotypes, generated from whole genome sequence, the major allele of all ASE SNP were classified as being either paternally or maternally derived. Not all SNP were able to be classified definitively. These SNP were used to construct a tissue by SNP major allele frequency heat map using the heat map function in the R software package gplots. To generate this heat map, if a SNP was classified as paternal, then the major allele frequency was unaltered, if the SNP was classified as maternal, then the major allele frequency was multiplied by −1. An additional tissue by tissue heat map was constructed by transposing a matrix of paternal allele frequencies then multiplying it by the original matrix of paternal allele frequencies.

To assign a parent-of-origin to genes, a custom C++ program was employed. For each gene, the program generated a ratio of parental classifications from all classified SNP within the gene. If this ratio was greater or equal to 0.75, the gene was classified as being either maternal or paternal. If the ratio was less than 0.75, the gene was classified as “undecided”. Excluding all “undecided” genes, a Wald-Wolfowitz runs test (R software package adehabitat, wawotest function) was performed for each tissue on an ordered list of classified genes to test if the patterns ofexpression, i.e. maternal or paternal, were significantly different from random, identifying regions that could be co-regulated or imprinted.

### ASE Validation dataset

Liver biopsies and blood samples were collected from the 20 first lactation Holstein cows on one occasion. Tissue collection was done with approval from the DEDJTR Animal Ethics Committee (application number 2011–23). Blood was collected by venipuncture of the coccygeal vein and was processed according to the blood fractionation and white blood cell stabilisation procedure in the RiboPure™ blood kit (Ambion by Life Technologies) protocol. White blood cells in RNA later were then stored at -20oC. Liver biopsies were collected by restraining cows in a crush and giving them 10 ml of lignocaine hydrochloride two percent into the subcutaneous, inter-costal and peritoneal tissues at the site of the insertion of the biopsy punch. A small incision was made with a scalpel before a biopsy punch was inserted into the liver to collectapproximately two to three grams of tissue. Following removal of the biopsy punch, betadine cream was placed in the incision site. Cows were given intramuscular antibiotics (Excenel RTU 2 ml/100 kg) and anti-inflammatory drugs (Ketoprofen 2 ml/100 kg) before being released from the crush. Immediately following collection samples were frozen in liquid nitrogen and then stored at −80 °C.

Total RNA was extracted from blood samples using the RiboPure™ Blood Kit (Ambion) and from liver samples using the RiboPure™ Kit (Ambion) as per manufacturer’s instructions. RNA quality was assessed on BioAnalyser 2100 (Agilent Technologies). RNAseq libraries were prepared using the TruSeq™ RNA Sample Preparation Kit v2 (Illumina) according to manufacturer’s instructions. Each library was randomly assigned to one of four pools and sequenced on a HiSeq™ 2000 (Illumina) in a 105 cycle paired end run. One hundred four base paired end reads were called with CASAVA v1.8 and output in fastq format. Sequence quality was assessed using FastQC. In house scripts were used to trim and filter poor quality bases and sequence reads. Bases with quality score less than 15 were trimmed from the end of reads. Reads with mean quality score less than 20, or greater than 3 N, or greater three consecutive bases had quality score less than 15, or final length less than 50 bases were discarded. Only paired reads were retained for alignment.

FImpute [41]was used to impute 28,899,038 SNP utilising the 1000 bull genomes project [10] Run 4.0 phased genotypes as reference population and either real or imputed high density (800 K) SNP genotypes on the individual animals. Where imputed high density SNP genotypes were imputed from BovineSNP50 data. Imputed and phased genotypes for each animal were generated by FImpute and then used to create parental genomes in the same way described above. Paired RNA reads were then aligned to each of the parental genomes using TOPHAT2 [34] with default input parameters.

Similar to the discovery dataset allele counts for each animals predicted heterozygous SNP that fell within annotated coding sequence were calculated and ASE tested using the Chi-squared (*χ*^*2*^) test outlined above. Also, a measure of reference allele mapping bias (BIAS) was calculated as

$$ BIAS=\sqrt{\frac{n_{A{P}_A}{n}_{B{P}_B}}{n_{A{P}_B}{n}_{B{P}_a}}} $$

where *n*_*AP*__A_ was the count of A alleles mapped to the parental genome with A allele, *n*_*BP*__B_ was the count of B alleles mapped to the parental genome with B allele, *n*_*AP*__B_ was the count of A alleles mapped to the parental genome with the B allele and *n*_*BP*__A_ was the count of B alleles mapped to the parental genome with A allele. To remove SNP that were likely to be imputation errors, i.e., predicted to be heterozygous when in fact the animal was homozygous for the UMD3.1 reference allele, and also those with significant reference allele mapping bias, SNP were filtered down to those that had reference allele mapping bias of less than two.

### Availability of supporting data

The data set, in the form of bam files, supporting the results of this article is available in the NCBI Sequence Read Archive, study accession number SRP042639.

## Additional files

Additional file 1: **Contains supplementary material including Figures S1 - S7, as well as Tables S1, S2, S6 - S9, S11 - S15.** (DOCX 7147 kb)

Additional file 2: **Contains supplementary material including Tables S3, S4, S5 and S10.** (XLSX 7470 kb)
