# Supplementary material for: Extensive variation between tissues in allele specific expression in an outbred mammal
Source: BMC Genomics. 2015 Nov 23;16:993. doi: 10.1186/s12864-015-2174-0 (PMC4657355; doi:10.1186/s12864-015-2174-0)
Supplement: Supplementary file 1 — Contains supplementary material including Figures S1 - S7, as well as Tables S1, S2, S6 - S9, S11 - S15. (DOCX 7147 kb) [file 12864_2015_2174_MOESM1_ESM.docx]

**
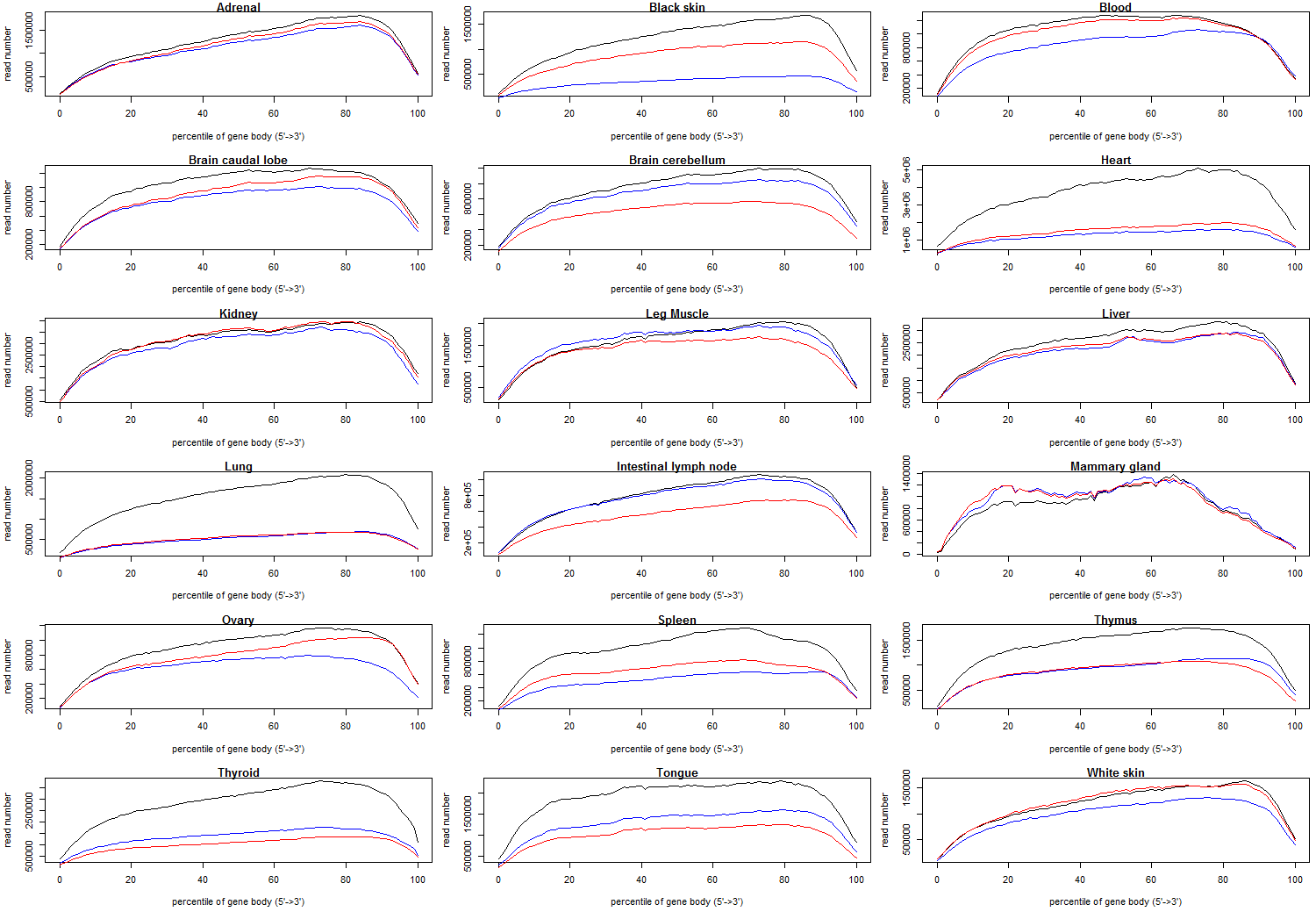
**

**Supplementary Figure 1.** Gene body plots for each tissue replicate performed using RNA-SeqQC. These are mean coverage plots for expressed transcripts from 5’ to 3’ end, with the lengths of transcripts normalized to 1-100.


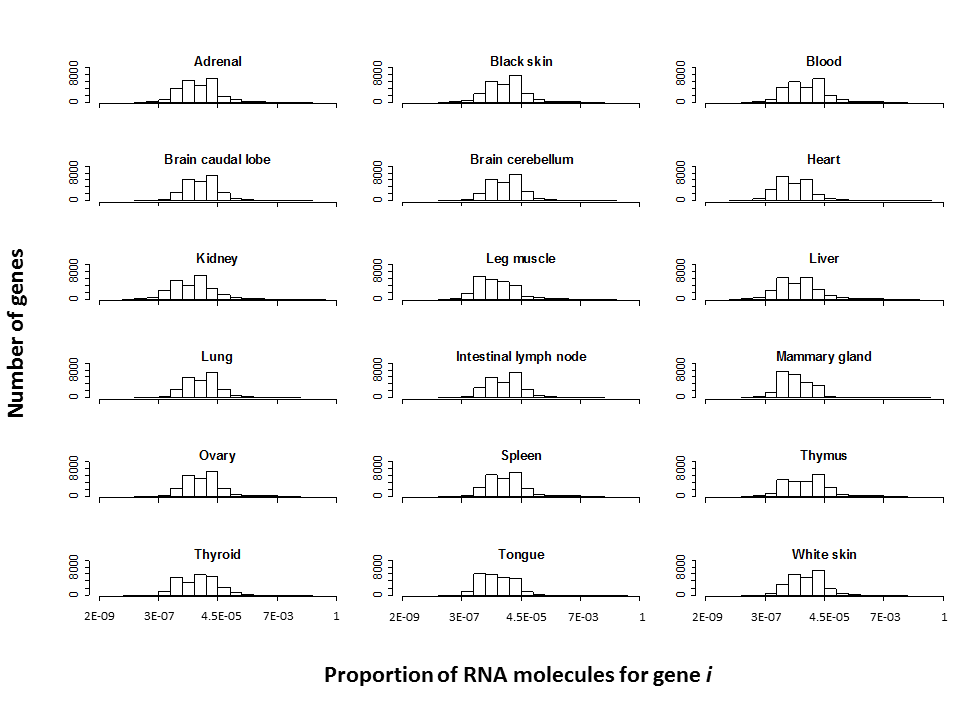


**Supplementary Figure 2.** The distribution of genes and the proportion of reads they contribute to the transcriptome.

**
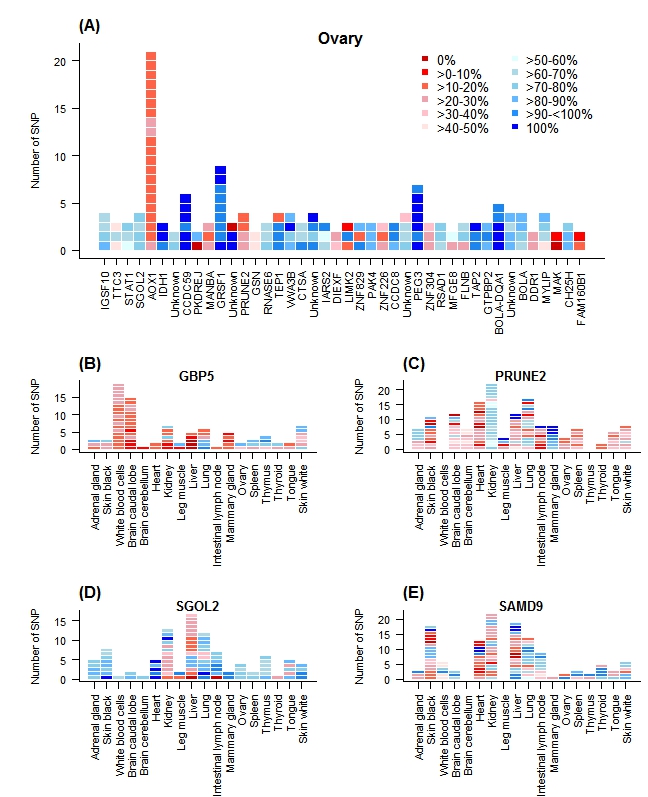
**

**Supplementary Figure 3.** Here each gene is shown as vertical bars of phased SNP tested within that gene, where red boxes are 0-50% paternal allele frequencies (maternal expression) and blue are 50-100% paternal allele frequencies (paternal expression). A) Displays 43 genes expressed in Ovary. B) Displays *GBP5* across all tissues. C) *PRUNE2* across all tissues D) *SGOL2* across all tissues and E) *SAMD9* across all tissues.


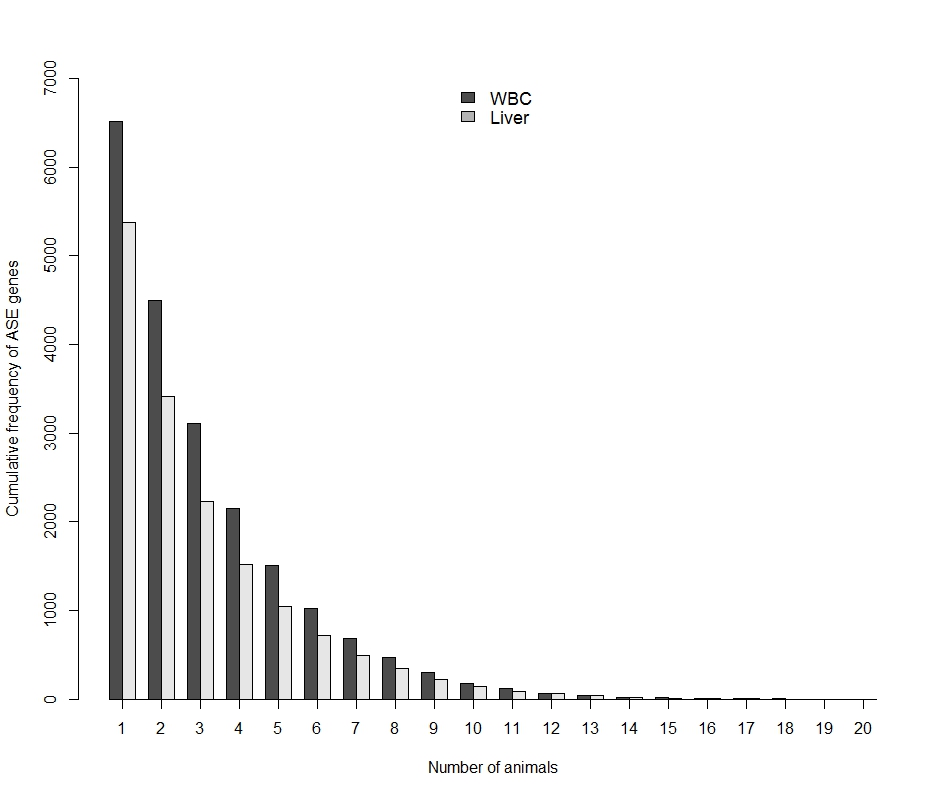


**Supplementary Figure 4.** A cumulative frequency histogram of the number of genes that showed significant ASE in one or more, through to all twenty animals in the validation dataset, for both WBC and liver.

**
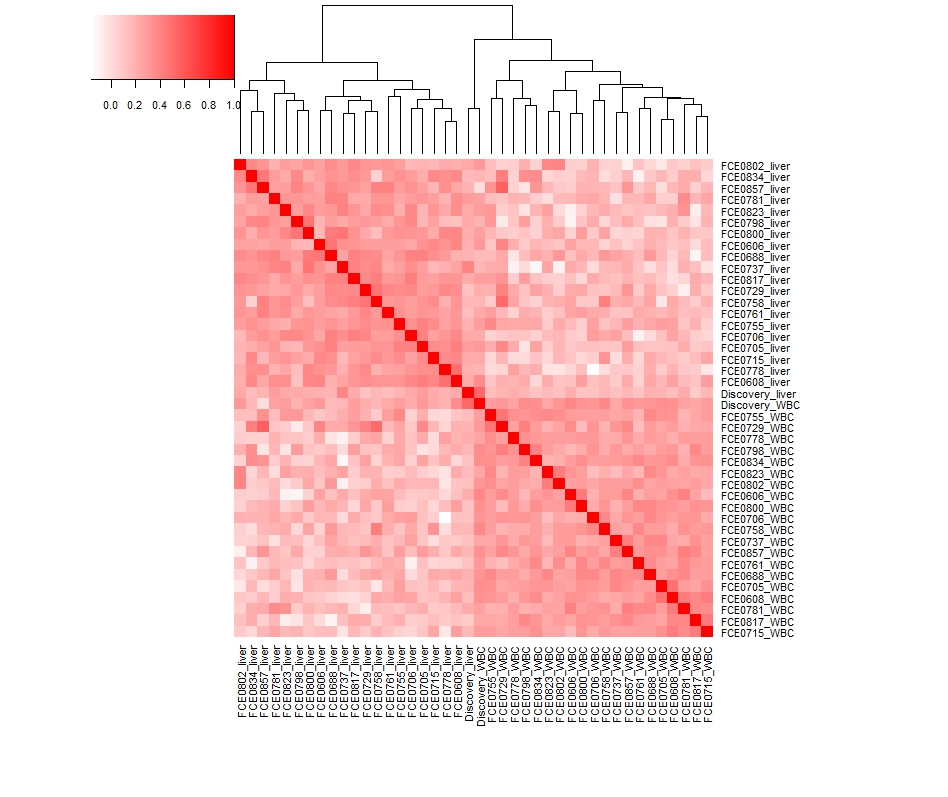
**

**Supplementary Figure 5.** Hierarchical clustering and heatmap of pairwise correlations of genes showing ASE between all samples, that is all animals (discovery and validation datasets) and all white blood cell and liver tissue samples. The variability between samples is measured by the height of the dendrogram branches. The colour key indicates the distance between samples with red being the least distant (or most correlated) and white being the most distant (or least correlated).


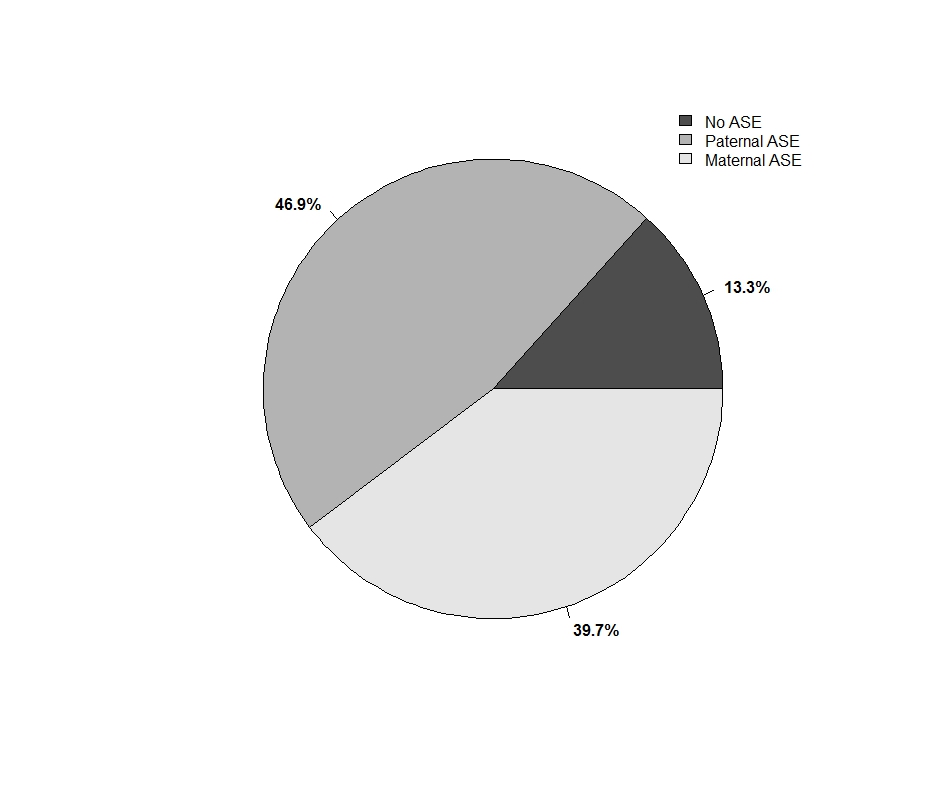


**Supplementary Figure 6.** A pie chart describing the average proportion of all genes, where parental origin could be established, that show biallelic (No ASE), maternal or paternal allele specific expression.


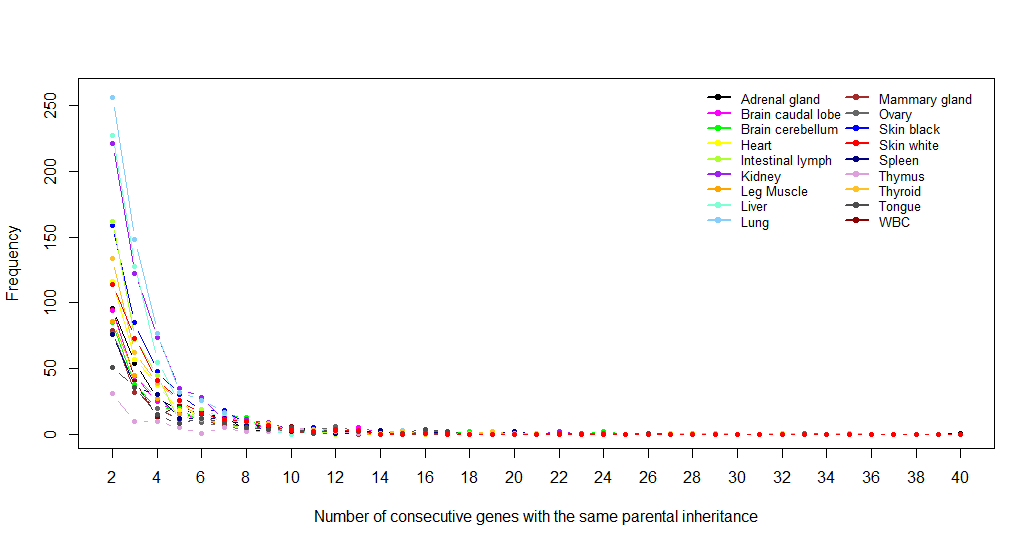


**Supplementary Figure 7.** Plot of the frequency of the number of consecutive genes with the same parental allele expressed in all 18 tissues.

**Supplementary Table 1** - Summary of allele specific expression studies to date, including their estimates of the extent of ASE, the species and number of samples (N) and which tissue was used, the method used to detect ASE and the number of genes tested.

| Publication | ASE (%) | Species | N | Tissue | Method | Genes Tested |
| --- | --- | --- | --- | --- | --- | --- |
| [[1](#_ENREF_1)] | 46 | Human | 96 |  |  | 13^*^ |
| [[2](#_ENREF_2)] | 46 | Human | 60 | Brain | Other | 15^*^ |
| [[3](#_ENREF_3)] | 54 | Human | 7 | Foetal | SNP array | 602^*^ |
| [[4](#_ENREF_4)] | 53 | Human | 12 | White blood cells | Microarray | 1389 |
| [[5](#_ENREF_5)] | 9.5^^^ | Human | 13 | LCL^&^ | SNP array | 3939 |
| [[6](#_ENREF_6)] | 68 | Human | 13 | Tumour cell lines | Other | 60^*^ |
| [[7](#_ENREF_7)] | 11 | Mice | 24 | Brain, liver, spleen | SNP array | 92^*^ |
| [[8](#_ENREF_8)] | 18 | Human | 210 | LCL | SNP array | 8233^$^ |
| [[9](#_ENREF_9)] | 22 | Human | 88 | LCL | SNP array | 1380 |
| [[10](#_ENREF_10)] | 10 | Human | 6 | LCL | Microarray | 12000^$^ |
| [[11](#_ENREF_11)] | 17 | Human | 67 | LCL | Microarray | 2635 |
| [[12](#_ENREF_12)] | 30 | Human | 53 | LCL | SNP array | 9751 |
| [[13](#_ENREF_13)] | 83 | Drosophila | 640 | Whole fly | Other | 18^*^ |
| [[14](#_ENREF_14)] |  | Arabidopsis | 2 | Seedlings | Microarray | 12311 |
| [[15](#_ENREF_15)] | 11-22 | Human | 8 | Cell lines | Other | 1789^$^ |
| [[16](#_ENREF_16)] | 18 | Human | 24 | Placenta | SNP array | 932^*^ |
| [[17](#_ENREF_17)] | 12 | Drosophila | 6 | Whole fly | RNAseq | 891 |
| [[18](#_ENREF_18)] | 5.7 | Mice | 1 | 52 brain tissues | RNAseq | 14520 |
| [[19](#_ENREF_19)] | 4.6 | Human | 4 | Primary CD4+ cells, blood# | RNAseq | 2701^$^ |
| [[20](#_ENREF_20)] | 51 | Drosophila | 14 | Whole fly | RNAseq | 9966 |
| [[21](#_ENREF_21)] | 54 | Human | 53 | LCL | SNP array | 755284^$^ |
| [[22](#_ENREF_22)] | 4 | Pigs | 2 | Gonad | RNAseq | 7572-11230^$^ |
| [[23](#_ENREF_23)] | 25 | Human | 180 | Varied | SAGE | 1295 |
| [[24](#_ENREF_24)] | 41 | Mouse | 1 | Liver, thymus, spleen, lung, hippocampus and heart | RNAseq | 6975 |
| [[25](#_ENREF_25)] | 89 | Human | 52 | Brain | Other | 74^*^ |
| [[26](#_ENREF_26)] | 37 | Drosophila | 6 | Whole fly | SNP array | 11929 |
| [[27](#_ENREF_27)] | 41 | Drosophila | 20 | Heads | RNAseq | 6369 |
| [[28](#_ENREF_28)] | 24 | Chickens | 12 | Spleen | RNAseq | 22655^$^ |
| [[29](#_ENREF_29)] | 30 | Human | 8 | Mammary epithelial cell lines | SNP array | 8779 |
| [[30](#_ENREF_30)] | 33 | Bovine | 5 | Blastocysts | RNAseq | 1018 |
| [[31](#_ENREF_31)] | 47 | Human | 46 |  | RNAseq | 2994 |
| [[32](#_ENREF_32)] | 6.5 | Human | 465 | LCL | RNAseq | 8420^$^ |
| [[33](#_ENREF_33)] | 89 | Mouse | 96 | Brain | RNAseq | 12682 |
| [[34](#_ENREF_34)] | 31 | Mouse | 8 | Liver, tail fibroblasts | RNAseq | 7465 |
| [[35](#_ENREF_35)] | 1.6-3.7 | Human | 175 | 29 solid organ tissues, 11 brain subregions, whole blood, LCL and skin fibroblast cells | RNAseq | 6385^$^ |

^^^monoallelic expression

^*^candidate gene studies

^#^only 1 tissue per individual i.e. cells or blood not both

^$^SNP tested not genes

^&^Lymphoblastoid cell lines (LCL)

**Supplementary Table 2.** Summary describing the number of raw read pairs generated per library, along with the number of read pairs passing QC, the percentage of reads aligned uniquely to the UMD3.1 reference for the TSE analysis and the percentage of reads aligned uniquely to the two parental genomes for the ASE analysis.

| Library | Millions of raw read pairs | Millions of read pairs pass QC | Millions uniquely aligned read pairs -UMD3.1 (% QC reads) | Millions uniquely aligned read pairs -Maternal (% QC reads) | Millions uniquely aligned read pairs - Paternal (% QC reads) |
| --- | --- | --- | --- | --- | --- |
| Adrenal1 | 21.6 | 18.4 | 17.2 (93%) | 15.7 (85%) | 15.8 (85%) |
| Adrenal2 | 17.9 | 15.4 | 14.3 (93%) | 12.6 (82%) | 12.6 (82%) |
| Adrenal3 | 21.3 | 18.1 | 16.8 (92%) | 15.1 (83%) | 15.2 (83%) |
| BrainCaudalLobe1 | 17.4 | 14.8 | 13.8 (93%) | 12.6 (85%) | 12.6 (85%) |
| BrainCaudalLobe2 | 15.0 | 12.8 | 12.0 (94%) | 10.8 (84%) | 10.8 (85%) |
| BrainCaudalLobe3 | 21.4 | 18.0 | 16.9 (93%) | 15.6 (86%) | 15.7 (87%) |
| BrainCerebellum1 | 21.4 | 18.3 | 17.1 (93%) | 15.9 (86%) | 15.9 (87%) |
| BrainCerebellum2 | 17.9 | 15.2 | 14.2 (93%) | 12.9 (85%) | 13.0 (85%) |
| BrainCerebellum3 | 13.7 | 11.5 | 10.8 (93%) | 10.0 (86%) | 10.0 (87%) |
| Heart1 | 15.0 | 12.8 | 11.4 (89%) | 7.18 (56%) | 7.17 (56%) |
| Heart2 | 12.1 | 10.6 | 9.69 (91%) | 5.90 (55%) | 5.87 (55%) |
| Heart3 | 38.8 | 33.0 | 29.9 (90%) | 19.4 (58%) | 19.3 (58%) |
| IntestinalLymph1 | 21.4 | 16.5 | 11.4 (69%) | 11.0 (67%) | 11.0 (67%) |
| IntestinalLymph2 | 19.7 | 15.3 | 12.9 (84%) | 12.6 (82%) | 12.5 (82%) |
| IntestinalLymph3 | 20.1 | 15.4 | 13.1 (85%) | 12.7 (83%) | 12.7 (82%) |
| Kidney1 | 41.1 | 35.1 | 32.4 (92%) | 26.8 (76%) | 26.7 (76%) |
| Kidney2 | 49.6 | 41.0 | 36.5 (89%) | 29.9 (72%) | 29.8 (72%) |
| Kidney3 | 48.4 | 40.8 | 37.7 (92%) | 31.1 (76%) | 31.0 (76%) |
| LegMuscle1 | 19.8 | 15.0 | 13.2 (88%) | 10.5 (70%) | 10.6 (70%) |
| LegMuscle2 | 23.8 | 18.1 | 15.9 (88%) | 12.9 (71%) | 12.9 (71%) |
| LegMuscle3 | 20.8 | 15.8 | 13.7 (87%) | 10.9 (69%) | 10.9 (69%) |
| Liver1 | 46.8 | 39.4 | 35.3 (89%) | 33.4 (84%) | 33.3 (84%) |
| Liver2 | 35.5 | 30.4 | 27.5 (90%) | 25.5 (84%) | 25.4 (83%) |
| Liver3 | 38.0 | 32.1 | 29.0 (90%) | 27.0 (84%) | 27.0 (84%) |
| Lung1 | 12.9 | 11.0 | 9.81 (88%) | 9.53 (86%) | 9.53 (86%) |
| Lung2 | 39.4 | 33.0 | 28.8 (87%) | 27.9 (84%) | 27.9 (84%) |
| Lung3 | 12.5 | 10.7 | 9.66 (89%) | 9.35 (87%) | 9.35 (87%) |
| Mammary1 | 15.2 | 11.8 | 9.81 (83%) | 10.1 (86%) | 10.1 (86%) |
| Mammary2 | 19.3 | 15.2 | 12.9 (84%) | 13.2 (86%) | 13.2 (86%) |
| Mammary3 | 17.9 | 13.8 | 11.7 (84%) | 12.0 (87%) | 12.0 (87%) |
| Ovary1 | 14.5 | 10.8 | 9.34 (86%) | 9.01 (83%) | 9.01 (83%) |
| Ovary2 | 21.5 | 16.8 | 14.4 (86%) | 13.9 (83%) | 13.9 (83%) |
| Ovary3 | 20.9 | 16.1 | 13.6 (84%) | 13.2 (82%) | 13.2 (82%) |
| SkinBlack1 | 6.7 | 6.1 | 5.61 (92%) | 5.46 (90%) | 5.46 (90%) |
| SkinBlack2 | 24.1 | 22.0 | 20.5 (93%) | 19.8 (90%) | 19.8 (90%) |
| SkinBlack3 | 16.6 | 15.0 | 14.0 (93%) | 13.6 (91%) | 13.6 (91%) |
| SkinWhite1 | 20.3 | 18.3 | 16.8 (92%) | 16.1 (88%) | 16.1 (88%) |
| SkinWhite2 | 18.7 | 16.9 | 15.6 (92%) | 15.1 (89%) | 15.1 (89%) |
| SkinWhite3 | 19.9 | 18.3 | 16.8 (92%) | 16.0 (87%) | 16.0 (87%) |
| Spleen1 | 17.3 | 11.6 | 9.22 (79%) | 8.95 (77%) | 8.94 (77%) |
| Spleen2 | 27.2 | 18.6 | 14.8 (79%) | 14.5 (78%) | 14.5 (78%) |
| Spleen3 | 16.3 | 11.2 | 8.69 (77%) | 8.49 (76%) | 8.48 (76%) |
| Thymus1 | 37.5 | 23.6 | 19.7 (83%) | 19.3 (82%) | 19.3 (81%) |
| Thymus2 | 49.2 | 31.8 | 26.0 (82%) | 26.0 (81%) | 26.0 (81%) |
| Thymus3 | 31.0 | 18.5 | 15.6 (84%) | 15.3 (83%) | 15.3 (83%) |
| Thyroid1 | 30.4 | 18.7 | 15.6 (83%) | 14.6 (78%) | 14.6 (78%) |
| Thyroid2 | 101.4 | 54.1 | 45.4 (84%) | 42.9 (79%) | 42.9 (79%) |
| Thyroid3 | 44.8 | 26.1 | 22.1 (85%) | 21.0 (80%) | 21.1 (80%) |
| Tongue1 | 35.3 | 22.0 | 18.7 (85%) | 14.0 (64%) | 14.0 (64%) |
| Tongue2 | 23.3 | 15.5 | 13.2 (85%) | 10.2 (66%) | 10.2 (65%) |
| Tongue3 | 19.8 | 12.1 | 10.3 (85%) | 7.98 (65%) | 7.97 (65%) |
| WBC1 | 17.3 | 14.7 | 13.5 (92%) | 13.1 (89%) | 13.1 (89%) |
| WBC2 | 16.1 | 13.9 | 12.8 (92%) | 12.4 (89%) | 12.4 (89%) |
| WBC3 | 19.6 | 16.7 | 15.4 (92%) | 15.0 (90%) | 14.9 (89%) |

**Supplementary Table 6.** To gain insight into where the variation in transcription was occurring a variance component analysis was performed. This table shows estimates of the variance accounted for by each term.

|  | Variance component | Proportion of variance explained |
| --- | --- | --- |
| gene | 0.71 | 0.34 |
| gene.tissue | 0.26 | 0.12 |
| gene.exon | 0.85 | 0.40 |
| error | 0.28 | 0.13 |

**Supplementary Table 7**. To gain insight into where the variation in transcription was occurring a variance component analysis was performed. This table shows solutions for exon_number_ for each tissue.

| Tissue | Solution |
| --- | --- |
| Overall | 1.216 |
| Adrenal gland | 0 |
| Brain caudal lobe | -0.260 |
| Brain cerebellum | -0.219 |
| Heart | -0.328 |
| Intestinal lymph | -0.051 |
| Kidney | -0.075 |
| Leg muscle | -0.394 |
| Liver | -0.194 |
| Lung | -0.098 |
| Mammary gland | -0.519 |
| Ovary | -0.168 |
| Skin black | -0.076 |
| Skin white | -0.043 |
| Spleen | -0.315 |
| Thymus | -0.144 |
| Thyroid | -0.076 |
| Tongue | -0.380 |
| White blood cells | -0.428 |

**Supplementary Table 8**. Mean reference (ref) allele frequencies where all SNP included (entire SNP set) or excluding SNP with reference allele frequency of 0 or 1 (reduced SNP set).

| Tissue | Mean ref AF  (Entire SNP set) | Mean ref AF (Reduced SNP set) |
| --- | --- | --- |
| Adrenal gland | 0.518 | 0.509 |
| Brain caudal lobe | 0.518 | 0.508 |
| Brain cerebellum | 0.515 | 0.506 |
| Heart | 0.522 | 0.508 |
| Intestinal lymph | 0.523 | 0.509 |
| Kidney | 0.516 | 0.508 |
| Leg muscle | 0.521 | 0.511 |
| Liver | 0.521 | 0.511 |
| Lung | 0.524 | 0.512 |
| Mammary gland | 0.517 | 0.509 |
| Ovary | 0.518 | 0.511 |
| Skin black | 0.514 | 0.506 |
| Skin white | 0.515 | 0.507 |
| Spleen | 0.517 | 0.508 |
| Thymus | 0.520 | 0.510 |
| Thyroid | 0.517 | 0.510 |
| Tongue | 0.517 | 0.508 |
| White blood cells | 0.521 | 0.509 |

**Supplementary Table 9**. Number of genes that show tissue specific ASE (TS ASE) exclusively in the tissues listed, as well as the number and proportion of those that show differential expression (DE) and the number and proportion of those DE that are up regulated in the tissue specific expression analysis.

| Tissue | Genes w/ exclusive TS ASE | Genes DE (%) | Genes up regulated (%) |
| --- | --- | --- | --- |
| Adrenal gland | 43 | 31 (72%) | 30 (96%) |
| Brain caudal lobe | 48 | 46 (95%) | 45 (97%) |
| Brain cerebellum | 57 | 49 (85%) | 49 (100%) |
| Heart | 36 | 25 (69%) | 24 (96%) |
| Intestinal lymph | 51 | 30 (58%) | 30 (100%) |
| Kidney | 172 | 110 (63%) | 103 (93%) |
| Leg Muscle | 21 | 12 (57%) | 12 (100%) |
| Liver | 156 | 117 (75%) | 108 (92%) |
| Lung | 224 | 121 (54%) | 115 (95%) |
| Mammary gland | 10 | 8 (80%) | 8 (100%) |
| Ovary | 32 | 25 (78%) | 25 (100%) |
| Skin black | 117 | 77 (65%) | 75 (97%) |
| Skin white | 70 | 50 (71%) | 49 (98%) |
| Spleen | 15 | 10 (66%) | 10 (100%) |
| Thymus | 11 | 11 (100%) | 11 (100%) |
| Thyroid | 65 | 34 (52%) | 33 (97%) |
| Tongue | 16 | 9 (56%) | 9 (100%) |
| WBC | 27 | 21 (77%) | 21 (100%) |

**Supplementary Table 11.** A table listing testable SNP (the chromosome and position) from the major milk protein genes in all 18 tissues along with the allele frequency of the major allele, where 0 is no ASE, 0.5 is 100% paternal expression and -0.5 is 100% maternal expression.

| **SNP (Chr_Position)** | **Gene Name** | **Adrenal gland** | **Brain caudal lobe** | **Brain cerebellum** | **Heart** | **Kidney** | **Leg muscle** | **Liver** | **Lung** | **Intestinal Lymph** | **Mammary gland** | **Ovary** | **Skin black** | **Skin white** | **Spleen** | **Thymus** | **Thyroid** | **Tongue** | **WBC** |
| --- | --- | --- | --- | --- | --- | --- | --- | --- | --- | --- | --- | --- | --- | --- | --- | --- | --- | --- | --- |
| 6_87280796 | CSN1S2 | 0 | 0.39 | 0.35 | 0 | 0 | 0 | 0 | 0 | 0.38 | 0 | 0 | 0 | 0 | 0 | 0 | 0.42 | 0.39 | 0 |
| 6_87280919 | CSN1S2 |  | 0.33 | 0.3 | 0 | 0 | 0 | 0 | 0 | 0.44 | 0 | 0 | 0 | 0 | 0 | 0 | 0 | 0.36 | 0 |
| 6_87181619 | CSN2 |  | 0.29 | 0.24 | 0 | -0.5 | 0 | 0 | 0 | 0.27 | 0 | 0 | 0 | 0 | 0 | 0 | 0 | 0.28 | 0 |

**Supplementary Table 12.** Summary describing the number of raw read pairs generated per library for the validation dataset, along with the number of read pairs passing QC and the percentage of reads aligned uniquely to the two parental genomes for the ASE analysis.

| Library | Millions of raw read pairs | Millions of read pairs pass QC (% raw reads) | Millions uniquely aligned read pairs -Maternal (% QC reads) | Millions uniquely aligned read pairs -Paternal (% QC reads) |
| --- | --- | --- | --- | --- |
| FCE0606-WBC | 18.8 | 14.3 (76.2%) | 12.4 (86.9%) | 12.4 (87.1%) |
| FCE0608-WBC | 21.4 | 16.8 (78.8%) | 15.0 (89.2%) | 15.0 (89.2%) |
| FCE0688-WBC | 19.8 | 17.6 (89.5%) | 15.5 (87.8%) | 15.5 (87.7%) |
| FCE0705-WBC | 24.2 | 18.4 (76.1%) | 15.9 (86.3%) | 15.9 (86.4%) |
| FCE0706-WBC | 20.9 | 16.1 (77.2%) | 13.9 (86.5%) | 13.9 (86.3%) |
| FCE0715-WBC | 20.4 | 15.6 (76.6%) | 13.9 (89.5%) | 13.9 (89.4%) |
| FCE0729-WBC | 12.8 | 11.4 (89.1%) | 10.0 (88.1%) | 10.0 (88.0%) |
| FCE0737-WBC | 26.0 | 20.0 (76.9%) | 17.3 (86.5%) | 17.3 (86.6%) |
| FCE0755-WBC | 16.2 | 14.8 (91.4%) | 13.0 (88.0%) | 13.0 (87.9%) |
| FCE0758-WBC | 16.5 | 12.5 (75.6%) | 9.90 (79.1%) | 9.89 (79.0%) |
| FCE0761-WBC | 22.1 | 16.9 (76.7%) | 14.5 (85.8%) | 14.5 (85.9%) |
| FCE0778-WBC | 26.2 | 23.3 (89.1%) | 20.4 (87.2%) | 20.3 (86.9%) |
| FCE0781-WBC | 18.3 | 14.1 (77.5%) | 12.6 (89.3%) | 12.6 (89.2%) |
| FCE0798-WBC | 21.5 | 19.1 (89.3%) | 16.8 (87.7%) | 16.8 (87.7%) |
| FCE0800-WBC | 20.2 | 14.9 (73.8%) | 12.9 (86.7%) | 12.9 (86.6%) |
| FCE0802-WBC | 21.0 | 15.5 (74.2%) | 13.4 (86.3%) | 13.4 (86.2%) |
| FCE0817-WBC | 17.8 | 13.5 (76.5%) | 11.7 (86.5%) | 11.7 (86.5%) |
| FCE0823-WBC | 21.8 | 19.4 (88.9%) | 16.8 (86.8%) | 16.8 (86.8%) |
| FCE0834-WBC | 18.7 | 16.6 (89.3%) | 14.5 (87.1%) | 14.5 (87.1%) |
| FCE0857-WBC | 20.0 | 15.2 (76.5%) | 13.6 (88.9%) | 13.5 (88.9%) |
| FCE0606-Liver | 17.0 | 13.2 (78.0%) | 12.0 (90.9%) | 12.0 (91.0%) |
| FCE0608-Liver | 20.1 | 18.0 (89.6%) | 16.4 (91.1%) | 16.4 (91.1%) |
| FCE0688-Liver | 18.4 | 14.0 (76.4%) | 12.8 (91.5%) | 12.8 (91.5%) |
| FCE0705-Liver | 19.3 | 16.8 (87.3%) | 15.2 (90.4%) | 15.2 (90.4%) |
| FCE0706-Liver | 16.0 | 14.5 (90.8%) | 13.2 (90.8%) | 13.2 (90.8%) |
| FCE0715-Liver | 17.1 | 13.1 (77.0%) | 11.9 (90.6%) | 11.9 (90.6%) |
| FCE0729-Liver | 16.2 | 14.5 (89.8%) | 13.4 (92.6%) | 13.4 (92.6%) |
| FCE0737-Liver | 11.6 | 10.5 (90.4%) | 9.74 (92.5%) | 9.73 (92.5%) |
| FCE0755-Liver | 20.8 | 18.4 (88.8%) | 16.8 (91.0%) | 16.8 (91.0%) |
| FCE0758-Liver | 13.0 | 11.8 (91.1%) | 10.9 (92.7%) | 10.9 (92.7%) |
| FCE0761-Liver | 26.6 | 23.6 (88.8%) | 20.8 (88.4%) | 20.8 (88.4%) |
| FCE0778-Liver | 19.0 | 14.3 (75.6%) | 13.1 (91.1%) | 13.1 (91.1%) |
| FCE0781-Liver | 16.3 | 12.4 (76.4%) | 11.3 (91.0%) | 11.3 (91.0%) |
| FCE0798-Liver | 17.7 | 13.6 (77.4%) | 12.5 (91.4%) | 12.5 (91.3%) |
| FCE0800-Liver | 24.3 | 21.7 (89.3%) | 19.8 (91.3%) | 19.8 (91.2%) |
| FCE0802-Liver | 17.0 | 13.2 (77.8%) | 11.8 (89.0%) | 11.7 (88.9%) |
| FCE0817-Liver | 16.9 | 14.7 (87.1%) | 13.6 (92.6%) | 13.6 (92.6%) |
| FCE0823-Liver | 14.6 | 13.3 (91.4%) | 12.3 (93.0%) | 12.3 (93.0%) |
| FCE0834-Liver | 14.8 | 13.5 (91.2%) | 12.3 (91.3%) | 12.3 (91.3%) |
| FCE0857-Liver | 14.8 | 11.4 (77.2%) | 10.4 (91.5%) | 10.4 (91.5%) |

**Supplementary Table 13.** Allele specific expression analysis results for a validation dataset of 20 first lactation dairy cows for white blood cells (WBC) and liver. The table contains the number of SNP tested and the number and proportion that showed significant ASE (ASE SNP) in each sample for each tissue, averaged across all samples within tissue (Average) and across all samples within tissue (Total). Also the number of genes containing SNP tested for ASE (Genes tested) and genes containing greater than one SNP tested for ASE (Genes w/ >1 SNP tested) and then the number and proportion that contained SNP significant for ASE (Genes w/ ASE SNP) and the number and proportion that contained greater than one SNP significant for ASE (Genes w/ >1 ASE SNP) in each sample for each tissue, averaged across all samples within tissue (Average) and across all samples within tissue (Total). Then finally the number and proportion of genes tested that showed significant ASE in at least one tissue but not both tissues tested (Genes w/ TS ASE SNP) in each sample for each tissue, averaged across all samples within tissue (Average) and across all samples within tissue (Total).

| Tissue | Sample | SNP tested | ASE SNP (% tested) | Genes tested | Genes w/ >1 SNP tested | Genes w/ ASE SNP  (% tested) | Genes w/ >1 ASE SNP  (% tested) | Genes w/ TS ASE SNP  (% tested) |
| --- | --- | --- | --- | --- | --- | --- | --- | --- |
| WBC | FCE0606 | 6,956 | 1,258 (18%) | 3,361 | 1,591 | 995 (29%) | 183 (11%) | 319 (9%) |
|  | FCE0608 | 8,771 | 1,723 (19%) | 3,617 | 1,933 | 1,190 (32%) | 310 (16%) | 358 (9%) |
|  | FCE0688 | 8,702 | 1,343 (15%) | 3,575 | 1,895 | 929 (25%) | 227 (11%) | 260 (7%) |
|  | FCE0705 | 8,130 | 1,519 (18%) | 3,694 | 1,848 | 1,109 (30%) | 259 (14%) | 405 (10%) |
|  | FCE0706 | 7,816 | 1,538 (19%) | 3,571 | 1,803 | 1,122 (31%) | 259 (14%) | 367 (10%) |
|  | FCE0715 | 10,347 | 1,833 (17%) | 4,086 | 2,261 | 1,263 (30%) | 343 (15%) | 296 (7%) |
|  | FCE0729 | 6,305 | 810 (12%) | 2,972 | 1,442 | 624 (20%) | 123 (8%) | 229 (7%) |
|  | FCE0737 | 8,102 | 1,671 (20%) | 3,639 | 1,811 | 1,219 (33%) | 291 (16%) | 320 (8%) |
|  | FCE0755 | 6,956 | 1,091 (15%) | 3,081 | 1,533 | 823 (26%) | 174 (11%) | 294 (9%) |
|  | FCE0758 | 8,696 | 1,449 (16%) | 3,620 | 1,937 | 1,076 (29%) | 237 (12%) | 319 (8%) |
|  | FCE0761 | 8,525 | 1,560 (18%) | 3,730 | 1,920 | 1,142 (30%) | 275 (14%) | 437 (11%) |
|  | FCE0778 | 7,564 | 1,323 (17%) | 3,405 | 1,690 | 969 (28%) | 221 (13%) | 282 (8%) |
|  | FCE0781 | 7,574 | 1,370 (18%) | 3,390 | 1,697 | 1,030 (30%) | 220 (12%) | 313 (9%) |
|  | FCE0798 | 8,522 | 1,380 (16%) | 3,561 | 1,893 | 974 (27%) | 233 (12%) | 288 (8%) |
|  | FCE0800 | 8,623 | 1,372 (15%) | 3,666 | 1,915 | 1,029 (28%) | 228 (11%) | 355 (9%) |
|  | FCE0802 | 7,060 | 1,284 (18%) | 3,317 | 1,623 | 992 (29%) | 191 (11%) | 341 (10%) |
|  | FCE0817 | 8,843 | 1,611 (18%) | 3,656 | 1,928 | 1,148 (31%) | 270 (14%) | 373 (10%) |
|  | FCE0823 | 7,035 | 1,219 (17%) | 3,148 | 1,588 | 908 (28%) | 199 (12%) | 262 (8%) |
|  | FCE0834 | 9,487 | 1,473 (15%) | 3,798 | 2,051 | 1,036 (27%) | 248 (12%) | 277 (7%) |
|  | FCE0857 | 8,677 | 1,511 (17%) | 3,728 | 1,923 | 1,126 (30%) | 258 (13%) | 301 (8%) |
|  | **Average** | **8,135** | **1,416 (17%)** | **3,531** | **1,814** | **1,035 (29%)** | **237 (13%)** | **319 (9%)** |
|  | **Totals** | **49,978** | **19,601 (39%)** | **8,970** | **6,298** | **6,521 (72%)** | **2,239 (35%)** | **3,072 (34%)** |
| Liver | FCE0606 | 5,204 | 884 (16%) | 2,599 | 1,145 | 642 (24%) | 132 (11%) | 241 (9%) |
|  | FCE0608 | 6,094 | 902 (14%) | 2,733 | 1,351 | 675 (24%) | 141 (10%) | 233 (8%) |
|  | FCE0688 | 5,885 | 1,055 (17%) | 2,788 | 1,301 | 777 (27%) | 159 (12%) | 316 (11%) |
|  | FCE0705 | 7,137 | 1,177 (16%) | 3,253 | 1,579 | 899 (27%) | 181 (11%) | 331 (10%) |
|  | FCE0706 | 6,744 | 1,037 (15%) | 3,011 | 1,456 | 719 (23%) | 159 (10%) | 221 (7%) |
|  | FCE0715 | 6,019 | 1,296 (21%) | 2,901 | 1,343 | 938 (32%) | 219 (16%) | 361 (12%) |
|  | FCE0729 | 7,650 | 1,376 (17%) | 3,180 | 1,612 | 916 (28%) | 217 (13%) | 312 (9%) |
|  | FCE0737 | 6,163 | 998 (16%) | 2,775 | 1,350 | 706 (25%) | 146 (10%) | 201 (7%) |
|  | FCE0755 | 6,441 | 1,012 (15%) | 2,934 | 1,370 | 781 (26%) | 154 (11%) | 318 (10%) |
|  | FCE0758 | 6,285 | 952 (15%) | 2,700 | 1,354 | 664 (24%) | 140 (10%) | 240 (8%) |
|  | FCE0761 | 8,663 | 1,523 (17%) | 3,500 | 1,811 | 1,008 (28%) | 255 (14%) | 383 (10%) |
|  | FCE0778 | 6,682 | 1,120 (16%) | 3,015 | 1,462 | 784 (26%) | 180 (12%) | 296 (9%) |
|  | FCE0781 | 6,819 | 1,173 (17%) | 2,916 | 1,410 | 777 (26%) | 187 (13%) | 270 (9%) |
|  | FCE0798 | 6,519 | 1,143 (17%) | 2,927 | 1,403 | 780 (26%) | 198 (14%) | 281 (9%) |
|  | FCE0800 | 6,981 | 1,231 (17%) | 3,152 | 1,540 | 906 (28%) | 196 (12%) | 349 (11%) |
|  | FCE0802 | 6,921 | 1,213 (17%) | 3,039 | 1,482 | 826 (27%) | 196 (13%) | 258 (8%) |
|  | FCE0817 | 7,091 | 1,151 (16%) | 3,076 | 1,504 | 853 (27%) | 169 (11%) | 320 (10%) |
|  | FCE0823 | 6,693 | 1,029 (15%) | 2,830 | 1,419 | 701 (24%) | 160 (11%) | 202 (7%) |
|  | FCE0834 | 5,998 | 914 (15%) | 2,777 | 1,322 | 666 (23%) | 145 (10%) | 260 (9%) |
|  | FCE0857 | 5,712 | 991 (17%) | 2,671 | 1,242 | 729 (27%) | 158 (12%) | 247 (9%) |
|  | **Average** | **6,585** | **1,108 (16%)** | **2,939** | **1,423** | **787 (26%)** | **174 (12%)** | **282 (9%)** |
|  | **Totals** | **40,093** | **15,201 (37%)** | **8,187** | **5,169** | **5,378 (65%)** | **1,624 (31%)** | **2,851 (34%)** |

**Supplementary Table 14.** Monoallelic expression results for a validation dataset of 20 first lactation dairy cows for white blood cells (WBC) and liver. The table contains the number and proportion of SNP tested that were showing monoallelic expression (MAE SNP), that is the major allele is at a frequency >90%, in each sample for each tissue, averaged across all samples within tissue (Average) and across all samples within tissue (Total). Also the number and proportion of genes tested that contained MAE SNP (Genes w/ MAE SNP). Then the number and proportion of genes with greater than one SNP showing MAE (Genes w/ 1 MAE SNP) in each sample for each tissue, averaged across all samples within tissue (Average) and across all samples within tissue (Total).

| Tissue | Sample | MAE SNP  (% tested) | Genes w/ MAE SNP  (% tested) | Genes w/ >1 MAE SNP  (% tested) |
| --- | --- | --- | --- | --- |
| WBC | FCE0606 | 139 (1.9%) | 130 (3.8%) | 8 (0.5%) |
|  | FCE0608 | 219 (2.4%) | 193 (5.3%) | 16 (0.8%) |
|  | FCE0688 | 152 (1.7%) | 133 (3.7%) | 13 (0.6%) |
|  | FCE0705 | 146 (1.7%) | 140 (3.7%) | 5 (0.2%) |
|  | FCE0706 | 175 (2.2%) | 159 (4.4%) | 10 (0.5%) |
|  | FCE0715 | 257 (2.4%) | 224 (5.4%) | 26 (1.1%) |
|  | FCE0729 | 116 (1.8%) | 104 (3.4%) | 7 (0.4%) |
|  | FCE0737 | 164 (2.0%) | 149 (4.0%) | 11 (0.6%) |
|  | FCE0755 | 127 (1.8%) | 122 (3.9%) | 4 (0.2%) |
|  | FCE0758 | 158 (1.8%) | 145 (4.0%) | 13 (0.6%) |
|  | FCE0761 | 156 (1.8%) | 143 (3.8%) | 11 (0.5%) |
|  | FCE0778 | 153 (2.0%) | 147 (4.3%) | 6 (0.3%) |
|  | FCE0781 | 163 (2.1%) | 151 (4.4%) | 11 (0.6%) |
|  | FCE0798 | 186 (2.1%) | 161 (4.5%) | 18 (0.9%) |
|  | FCE0800 | 177 (2.0%) | 160 (4.3%) | 12 (0.6%) |
|  | FCE0802 | 123 (1.7%) | 119 (3.5%) | 3 (0.1%) |
|  | FCE0817 | 189 (2.1%) | 173 (4.7%) | 9 (0.4%) |
|  | FCE0823 | 152 (2.1%) | 137 (4.3%) | 13 (0.8%) |
|  | FCE0834 | 194 (2.0%) | 175 (4.6%) | 12 (0.5%) |
|  | FCE0857 | 190 (2.1%) | 172 (4.6%) | 13 (0.6%) |
|  | **Average** | **166 (2.0%)** | **151 (4.3%)** | **11 (0.6%)** |
|  | **Totals** | **2,823 (5.6%)** | **1,989 (22%)** | **159 (2.5%)** |
| Liver | FCE0606 | 90 (1.7%) | 77 (2.9%) | 7 (0.6%) |
|  | FCE0608 | 107 (1.7%) | 96 (3.5%) | 8 (0.5%) |
|  | FCE0688 | 109 (1.8%) | 104 (3.7%) | 4 (0.3%) |
|  | FCE0705 | 140 (1.9%) | 127 (3.9%) | 10 (0.6%) |
|  | FCE0706 | 131 (1.9%) | 118 (3.9%) | 7 (0.4%) |
|  | FCE0715 | 150 (2.4%) | 125 (4.3%) | 14 (1.0%) |
|  | FCE0729 | 182 (2.3%) | 157 (4.9%) | 16 (0.9%) |
|  | FCE0737 | 122 (1.9%) | 110 (3.9%) | 9 (0.6%) |
|  | FCE0755 | 120 (1.8%) | 111 (3.7%) | 8 (0.5%) |
|  | FCE0758 | 133 (2.1%) | 112 (4.1%) | 15 (1.1%) |
|  | FCE0761 | 189 (2.1%) | 161 (4.6%) | 17 (0.9%) |
|  | FCE0778 | 132 (1.9%) | 116 (3.8%) | 12 (0.8%) |
|  | FCE0781 | 149 (2.1%) | 121 (4.1%) | 12 (0.8%) |
|  | FCE0798 | 123 (1.8%) | 105 (3.5%) | 12 (0.8%) |
|  | FCE0800 | 151 (2.1%) | 142 (4.5%) | 9 (0.5%) |
|  | FCE0802 | 158 (2.2%) | 133 (4.3%) | 12 (0.8%) |
|  | FCE0817 | 149 (2.1%) | 139 (4.5%) | 8 (0.5%) |
|  | FCE0823 | 148 (2.2%) | 128 (4.5%) | 12 (0.8%) |
|  | FCE0834 | 100 (1.6%) | 94 (3.3%) | 6 (0.4%) |
|  | FCE0857 | 144 (2.5%) | 122 (4.5%) | 11 (0.8%) |
|  | **Average** | **136 (2.0%)** | **119 (4.0%)** | **10 (0.7%)** |
|  | **Totals** | **2,358 (5.8%)** | **1,638 (20%)** | **136 (2.6%)** |

**Supplementary Table 15.** A table listing the 17 imprinted genes, the allele that was expressed in this dataset, Paternal (P), Maternal (M) or TS specifies that the expressed allele was tissue specific. Also whether they were Not imprinted (N), Imprinted (I), or Partially imprinted (P) for each of the 18 tissues. Where the cell is empty the gene was not expressed or the coverage of the SNP was less than 10x. For the genes with tissue specific expression, the superscript M, P and T represent the maternal, paternal or transcript specific ASE respectively.

| Gene Name | Expressed Allele | Adrenal | Black skin | Blood | Brain caudal lobe | Brain cerebellum | Heart | Kidney | Leg muscle | Liver | Lung | Intestinal Lymph | Mammary | Ovary | Spleen | Thymus | Thyroid | Tongue | White skin |
| --- | --- | --- | --- | --- | --- | --- | --- | --- | --- | --- | --- | --- | --- | --- | --- | --- | --- | --- | --- |
| *NAP1L5* |  | I |  | I | I | I | I | I |  | I | I |  |  | I | I | I | I |  | I |
| *DLX5* | P |  |  |  |  |  |  | I |  |  |  |  |  |  |  |  |  |  |  |
| *SLC22A3* | M | I |  |  |  | I | I | I |  |  |  |  |  | I |  |  |  |  |  |
| *RTL1* | P | I |  |  |  |  |  |  |  |  |  |  |  |  |  |  |  |  |  |
| *NLRP2* | P |  |  | P |  |  |  |  |  |  |  |  |  |  |  |  |  |  |  |
| *Igf2r* | M | N |  | N | N | N |  | P | I | N | I | N |  | I | N |  | N | I | N |
| *Pon2* | P | N | N | N | N | N | N | P | N | N | N |  |  |  | N |  | N | N | N |
| *Igf2* | P | I | N |  | I | I | I | I |  | I |  | I |  | I | I |  | I | I | I |
| *COPG2IT1* | P | N | N | N | N | N |  | N | N |  | I |  |  | N | N | N | N | N | N |
| *PPP1R9A* | P | N |  |  | N | N |  | I |  |  | I |  |  | N | N |  | N | N |  |
| *ATP10A* | P | N | P |  | N | N |  | P |  |  | P |  |  | P |  |  | N |  | N |
| *Pon3* | P | I | N |  |  | I | I | N | N | N | N |  | I |  | I |  | N | N | N |
| *Gab1* | P | N | N | N | N | N | N | N | N | N | N | N | P | N | N | N | N | N | N |
| *Impact* | P | P | N | P | P | P | P | P | P | P | P | P | P | P | P | P | P | P | P |
| *RB1* | P | P | P | N | N | N | N | N | N | P | N | P | N | N | N | N | N | N | N |
| *Ampd3* | TS | N |  | N | N |  | N | N | N |  | P_M_ | P_P_ |  |  | N |  | P_P_ | N | N |
| *GRB10* | P | P | P |  | N | N | P | N | P | P | P | P | P | P | P |  | N | P | N |

# References

1. Yan H, Yuan W, Velculescu VE, Vogelstein B, Kinzler KW: **Allelic variation in human gene expression.** *Science* 2002, **297:**1143.

2. Bray NJ, Buckland PR, Owen MJ, O'Donovan MC: **Cis-acting variation in the expression of a high proportion of genes in human brain.** *Human Genetics* 2003, **113:**149-153.

3. Lo HS, Wang Z, Hu Y, Yang HH, Gere S, Buetow KH, Lee MP: **Allelic variation in gene expression is common in the human genome.** *Genome Research* 2003, **13:**1855-1862.

4. Pant PVK, Tao H, Beilharz EJ, Ballinger DG, Cox DR, Frazer KA: **Analysis of allelic differential expression in human white blood cells.** *Genome Research* 2006, **16:**331-339.

5. Gimelbrant A, Hutchinson JN, Thompson BR, Chess A: **Widespread monoallelic expression on human autosomes.** *Science* 2007, **318:**1136-1140.

6. Milani L, Gupta M, Andersen M, Dhar S, Fryknäs M, Isaksson A, Larsson R, Syvänen AC: **Allelic imbalance in gene expression as a guide to cis-acting regulatory single nucleotide polymorphisms in cancer cells.** *Nucleic Acids Research* 2007, **35**.

7. Campbell CD, Kirby A, Nemesh J, Daly MJ, Hirschhorn JN: **A survey of allelic imbalance in F1 mice.** *Genome Research* 2008, **18:**555-563.

8. Dimas AS, Stranger BE, Beazley C, Finn RD, Ingle CE, Forrest MS, Ritchie ME, Deloukas P, Tavaré S, Dermitzakis ET: **Modifier effects between regulatory and protein-coding variation.** *Plos Genetics* 2008, **4**.

9. Serre D, Gurd S, Ge B, Sladek R, Sinnett D, Harmsen E, Bibikova M, Chudin E, Barker DL, Dickinson T, et al: **Differential allelic expression in the human genome: A robust approach to identify genetic and epigenetic Cis-acting mechanisms regulating gene expression.** *Plos Genetics* 2008, **4**.

10. Bjornsson HT, Albert TJ, Ladd-Acosta CM, Green RD, Rongione MA, Middle CM, Irizarry RA, Broman KW, Feinberg AP: **SNP-specific array-based allele-specific expression analysis.** *Genome Research* 2008, **18:**771-779.

11. Pollard KS, Serre D, Wang X, Tao H, Grundberg E, Hudson TJ, Clark AG, Frazer K: **A genome-wide approach to identifying novel-imprinted genes.** *Human Genetics* 2008, **122:**625-634.

12. Ge B, Pokholok DK, Kwan T, Grundberg E, Morcos L, Verlaan DJ, Le J, Koka V, Lam KCL, Gagné V, et al: **Global patterns of cis variation in human cells revealed by high-density allelic expression analysis.** *Nature Genetics* 2009, **41:**1216-1222.

13. Gruber JD, Long AD: **Cis-regulatory variation is typically polyallelic in Drosophila.** *Genetics* 2009, **181:**661-670.

14. Zhang X, Borevitz JO: **Global analysis of allele-specific expression in Arabidopsis thaliana.** *Genetics* 2009, **182:**943-954.

15. Zhang K, Li JB, Gao Y, Egli D, Xie B, Deng J, Li Z, Lee JH, Aach J, Leproust EM, et al: **Digital RNA allelotyping reveals tissue-specific and allele-specific gene expression in human.** *Nature Methods* 2009, **6:**613-618.

16. Daelemans C, Ritchie ME, Smits G, Abu-Amero S, Sudbery IM, Forrest MS, Campino S, Clark TG, Stanier P, Kwiatkowski D, et al: **High-throughput analysis of candidate imprinted genes and allele-specific gene expression in the human term placenta.** *BMC Genetics* 2010, **11:**25.

17. Fontanillas P, Landry CR, Wittkopp PJ, Russ C, Gruber JD, Nusbaum C, Hartl DL: **Key considerations for measuring allelic expression on a genomic scale using high-throughput sequencing.** *Molecular Ecology* 2010, **19:**212-227.

18. Gregg C, Zhang J, Weissbourd B, Luo S, Schroth GP, Haig D, Dulac C: **High-resolution analysis of parent-of-origin allelic expression in the mouse brain.** *Science* 2010, **329:**643-648.

19. Heap GA, Yang JHM, Downes K, Healy BC, Hunt KA, Bockett N, Franke L, Dubois PC, Mein CA, Dobson RJ, et al: **Genome-wide analysis of allelic expression imbalance in human primary cells by high-throughput transcriptome resequencing.** *Human Molecular Genetics* 2010, **19:**122-134.

20. McManus CJ, Coolon JD, Duff MO, Eipper-Mains J, Graveley BR, Wittkopp PJ: **Regulatory divergence in Drosophila revealed by mRNA-seq.** *Genome Research* 2010, **20:**816-825.

21. Wagner JR, Ge B, Pokholok D, Gunderson KL, Pastinen T, Blanchette M: **Computational analysis of whole-genome differential allelic expression data in human.** *PLoS Computational Biology* 2010, **6:**24.

22. Esteve-Codina A, Kofler R, Palmieri N, Bussotti G, Notredame C, Pérez-Enciso M: **Exploring the gonad transcriptome of two extreme male pigs with RNA-seq.** *Bmc Genomics* 2011, **12**.

23. Vidal DO, De Souza JES, Pires LC, Masotti C, Salim ACM, Costa MCF, Galante PAF, De Souza SJ, Camargo AA: **Analysis of allelic differential expression in the human genome using allele-specific serial analysis of gene expression tags.** *Genome* 2011, **54:**120-127.

24. Keane TM, Goodstadt L, Danecek P, White MA, Wong K, Yalcin B, Heger A, Agam A, Slater G, Goodson M, et al: **Mouse genomic variation and its effect on phenotypes and gene regulation.** *Nature* 2011, **477:**289-294.

25. Xu X, Wang H, Zhu M, Sun Y, Tao Y, He Q, Wang J, Chen L, Saffen D: **Next-generation DNA sequencing-based assay for measuring allelic expression imbalance (AEI) of candidate neuropsychiatric disorder genes in human brain.** *Bmc Genomics* 2011, **12:**518.

26. Yang Y, Graze RM, Walts BM, Lopez CM, Baker HV, Wayne ML, Nuzhdin SV, McIntyre LM: **Partitioning transcript variation in drosophila: Abundance, isoforms, and alleles.** *G3: Genes, Genomes, Genetics* 2011, **1:**427-436.

27. Graze RM, Novelo LL, Amin V, Fear JM, Casella G, Nuzhdin SV, McIntyre LM: **Allelic imbalance in drosophila hybrid heads: Exons, isoforms, and evolution.** *Molecular Biology and Evolution* 2012, **29:**1521-1532.

28. MacEachern S, Muir WM, Crosby SD, Cheng HH: **Genome-wide identification and quantification of cis- and trans-regulated genes responding to Marek's disease virus infection via analysis of allele-specific expression.** *Frontiers in Genetics* 2012, **2**.

29. Gao C, Devarajan K, Zhou Y, Slater CM, Daly MB, Chen X: **Identifying breast cancer risk loci by global differential allele-specific expression (DASE) analysis in mammary epithelial transcriptome.** *Bmc Genomics* 2012, **13:**570.

30. Chitwood JL, Rincon G, Kaiser GG, Medrano JF, Ross PJ: **RNA-seq analysis of single bovine blastocysts.** *Bmc Genomics* 2013, **14:**350.

31. Zhang S, Wang F, Wang H, Zhang F, Xu B, Li X, Wang Y: **Genome-wide identification of allele-specific effects on gene expression for single and multiple individuals.** *Gene* 2014, **533:**366-373.

32. Lappalainen T, Sammeth M, Friedländer MR, T Hoen PAC, Monlong J, Rivas MA, Gonzàlez-Porta M, Kurbatova N, Griebel T, Ferreira PG, et al: **Transcriptome and genome sequencing uncovers functional variation in humans.** *Nature* 2013, **501:**506-511.

33. Crowley JJ, Zhabotynsky V, Sun W, Huang S, Pakatci IK, Kim Y, Wang JR, Morgan AP, Calaway JD, Aylor DL, et al: **Analyses of allele-specific gene expression in highly divergent mouse crosses identifies pervasive allelic imbalance.** *Nature Genetics* 2015, **Advanced online article**.

34. Pinter SF, Colognori D, Beliveau BJ, Sadreyev RI, Payer B, Yildirim E, Wu C-t, Lee JT: **Allelic imbalance is a prevalent and tissue-specific feature of the mouse transcriptome.** *Genetics* 2015, **200:**ahead of print.

35. GTEx Consortium: **The Genotype-Tissue Expression (GTEx) pilot analysis: Multitissue gene regulation in humans.** *Science* 2015, **348:**648-660.
